# Scalable and cost-efficient custom gene library assembly from oligopools

**DOI:** 10.1126/sciadv.ady2279

**Published:** 2026-05-22

**Authors:** Chase R. Freschlin, Kevin K. Yang, Philip A. Romero

**Affiliations:** ^1^ Department of Biochemistry, University of Wisconsin–Madison, Madison, WI, USA.; ^2^ Microsoft Research, Cambridge, MA, USA.; ^3^ Department of Biomedical Engineering, Duke University, Durham, NC, USA.

## Abstract

Advances in metagenomics, deep learning, and generative protein design have enabled broad in silico exploration of sequence space, but experimental characterization is still constrained by the cost and scalability of DNA synthesis. Here, we present OMEGA (Oligo-based Multiplexed Efficient Gene Assembly), a low-cost, accessible method for assembling hundreds to thousands of full-length genes in parallel using standard laboratory techniques. OMEGA computationally fragments target genes into short, high-fidelity Golden Gate–compatible oligonucleotides that can be ordered as a pooled library and assembled across multiplexed subpools. We systematically optimized the number of fragments per gene and orthogonal ligation sites per reaction and determine that OMEGA can assemble up to 2.6-kilobase constructs using as many as 70 Golden Gate sites. To validate the approach, we assembled and functionally screened a library of 810 natural and synthetic green fluorescent protein variants, recovering 94 to 97% of target sequences with high uniformity. OMEGA enables precision library construction at scale, with per-gene costs as low as $1.50, and offers a broadly applicable solution for bridging computational protein design with high-throughput experimental validation. We have developed OMEGA as an open-source software package and an easy-to-use Colab notebook to facilitate community adaptation.

## Teaser

OMEGA enables scalable, low-cost construction of large gene libraries using multiplexed Golden Gate assembly.

## INTRODUCTION

The explosion of biological data, coupled with advances in deep learning and generative artificial intelligence, has transformed protein engineering into a data-driven discipline. We have unlocked vast sequence diversity with advancements in large-scale metagenomic sequencing (*1*, *2*) and generated detailed sequence-function landscapes with high-throughput data generation (*3*–*5*). These data have given us the power to design limitless mutants, synthetic homologs, and de novo designs in silico that explore vast swathes of sequence space (*6*–*9*). However, the ability to experimentally characterize these designs remains a major bottleneck. Scalable gene synthesis methods are essential to bridge this gap, enabling targeted exploration of sequence space with the same precision as data-driven models to expand our knowledge of protein function.

Current DNA synthesis methods are constrained by a strict trade-off between sequence length and scale that limits the scope of precision libraries. To synthesize custom sequences, researchers must choose between synthesizing a few long sequences as gene fragments ≤5000 base pairs (bp) or many short sequences as oligonucleotide pools (oligopools; ~300 bp) from manufacturers like Twist Bioscience and Integrated DNA Technologies (IDT). Oligopools are heterogeneous mixes of single-stranded DNA where each strand is uniquely specified by the user (*10*). They are substantially cheaper than fragment synthesis per base, but constrained to DNA fragments shorter than most protein coding genes.

We can expand the encodable length of oligopools by using DNA assembly to build larger constructs from multiple oligos. Scaling DNA assembly for large, diverse sequence libraries presents challenges in workflow complexity, specialized equipment needs, assembly accuracy, and construct length. For example, polymerase cycling assembly (PCA) can stitch together multiple oligos in individual reactions but struggles when scaled to many highly similar sequences (*11*–*15*). High-throughput PCA assemblies using DropSynth (*16*) resulted in low-fidelity libraries that contained ~80% of target genetic constructs with a high background of incorrect products. Alternatively, Golden Gate (GG) cloning uses Type IIS restriction enzymes to generate unique DNA overhangs called GG sites (commonly 4 bp), which allows for efficient assembly of multiple DNA fragments (*17*) Data-optimized assembly design (DAD) identifies combinations of GG sites that yield high-fidelity DNA assemblies, allowing for high-complexity assemblies using tens of GG sites, and have enabled the assembly of large constructs directly from oligopools (*18*–*20*). In theory, this approach can be applied to assemble multiple pooled constructs from a single GG reaction if all assembly sites are sufficiently orthogonal.

In this work, we present a low-cost and accessible method for precisely assembling hundreds to thousands of genes in parallel using standard molecular biology techniques and equipment. Our method computationally splits a list of target genes into fragments optimized for high-fidelity GG assembly and that can be ordered as an oligopool. The oligopool is divided into several subpools in a microtiter plate, where several genes are assembled per pool, yielding hundreds of precisely specified genes. To develop this method, we systematically optimized key DNA assembly parameters, including the number of GG sites per subassembly and the number of ligated fragments, to assess scalability across gene lengths and library sizes. We demonstrated the scalability of our approach by assembling a diverse panel of 810 natural and designed green fluorescent protein (GFP) genes, achieving 94 to 97% recovery of target genes. The final gene library was highly uniform with 87 to 92% of genes within 10-fold of the median-abundant sequence. We performed downstream functional screening of the designed GFP variants to demonstrate the quality of the assembled genes. Our method requires only standard laboratory equipment, minimal hands-on time, and takes 2 days from start to finish, enabling the cost-effective assembly of hundreds to thousands of genes for $1.50 to $14 per gene for constructs up to 2.6 kb.

## RESULTS

### OMEGA: Oligo-based Multiplexed Efficient Gene Assembly

**Fig. 1.**
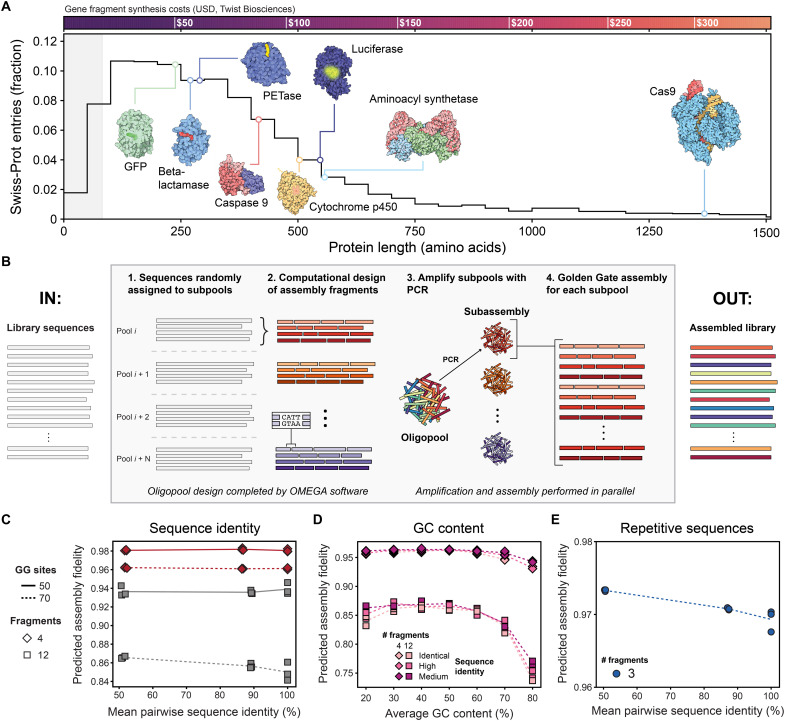
Oligo-based Multiplexed Efficient Gene Assembly (OMEGA). (**A**) Distribution of protein lengths for all entries in Swiss-Prot. Most proteins are too large to encode on single oligos, represented as the shaded gray area. Oligos provide ~250 bp of coding sequence given a 300-bp oligo with PCR and cloning adaptors. The synthesis cost per gene is shown above the distribution using Twist Bioscience prices for fragment synthesis. Structures are from RCSB Molecule of the Month articles and use the following structure codes from left to right: 1GFL, 4EYL, 5XH3, 1NW9, 1W0E, 2D1S, 1ASZ, and 4OO8. USD, US dollar. (**B**) OMEGA design and assembly workflow. Users provide a list of codon-optimized sequences that OMEGA randomly sorts into *N* subassembly pools. For each subpool, OMEGA designs fragments that use high-fidelity GG sites and ensures that all pieces are within oligo size constraints. Subassembly pools are separately amplified and assembled into full-length gene products in parallel, after which assembly products are combined to recover the designed library. (**C**) Effect of sequence identity on assembly fidelity. Average predicted assembly fidelity for subpools containing sequences with 50, ~88, or 100% pairwise identity. Each condition was tested using assemblies of 4 or 12 fragments and subpools containing 50 or 70 GG sites. (**D**) Effect of GC content on assembly fidelity. Average predicted assembly fidelity for synthetic sequences with GC content ranging from 20 to 80%. Assemblies were tested for both 4- and 12-fragment constructs and across varied sequence identities. (**E**) Assembly fidelity of repetitive sequences. Average predicted assembly fidelity for DARPin sequences composed of five ankyrin repeats, using 70 GG sites per subpool and varying sequence identities. Mean fidelities across all subpools differed by less than 1%.

Most proteins, including many with applications in medicine and chemistry, require individual synthesis as gene fragments because their genes are too long to fit on a single oligonucleotide (Fig. 1A). Proteins such as PETases, luciferases, and Cas9, which can be engineered to recycle plastics, image in vivo processes, and edit genomes, cost more than $50 per variant to synthesize, making large-scale experiments impractical. There is substantial interest and need for new methods to assemble DNA fragments from oligopools into thousands of gene-length sequences (*21*).

We set out to develop a simple and accessible method for assembling hundreds to thousands of user-defined genes from oligopools, reducing per-gene synthesis costs. A key strategy for scaling gene assembly is multiplexing, in which multiple genes are assembled simultaneously in a single reaction. Previous studies have shown that GG cloning, when paired with precisely designed assembly sites, can reliably assemble large DNA constructs up to 40 kb from as many as 52 fragments. This is made possible by DAD, which uses empirical ligation data from all combinations of 4-bp overhangs to identify sets of high-fidelity 4-bp overhangs that predominantly ligate with their correct partners and minimize off-target cross-talk. We reasoned that this same principle could be adapted for multiplexed assembly of many smaller (e.g., ~1 kb) genes rather than one large construct. For example, a single 1-kb gene can be assembled from five 300-bp oligos using four unique GG sites between each oligo and two universal terminal GG sites that insert the assembled product into a vector. A multiplexed assembly using 50 GG sites would theoretically be able to assemble twelve 1-kb genes in a single reaction from 60 oligos.

We present Oligo-based Multiplexed Efficient Gene Assembly (OMEGA) as a hierarchical gene assembly method that enables scalable construction of large gene libraries (Fig. 1B). The process begins with an oligopool that is divided into a maximum of 92 subpools using polymerase chain reaction (PCR) with unique primer pairs from a set of experimentally verified, orthogonal primers (*22*). Within each subpool, up to 70 genes are assembled simultaneously using multiplexed GG cloning. The central design challenge is determining how to create the input oligopool so that all gene fragments can be reliably assembled into their intended genes without cross-talk. OMEGA takes as input a list of nucleotide sequences and the maximum number of GG sites allowed per subpool assembly. The number of oligos used to encode each gene is calculated from the longest library sequence to mitigate variable assembly efficiencies across constructs and determines the number of GG sites needed for each gene. The specified maximum number of GG sites per subpool then dictates how many genes can be multiplexed together in a single assembly reaction. Last, OMEGA determines how many subpools are required to cover all input genes and randomly assigns genes to each subpool.

Within each subpool, OMEGA must identify a set of fragmentation sites—specific 4-bp regions that define the junctions between adjacent oligos. These sites must be mutually orthogonal, meaning each overhang pairs only with its correct complement and not with any other site in the subpool. The assembly fidelity of a given set of sites is calculated as the product of their individual ligation probabilities, which represents the likelihood that all overhangs ligate correctly in the presence of one another. We formulate gene fragmentation as a combinatorial optimization problem with the objective of identifying fragmentation sites that maximize assembly fidelity while satisfying constraints on oligo length. We used simulated annealing (SA), starting with sites placed uniformly across the genes and iteratively swapping/accepting new sites according to SA criteria. We run multiple independent optimization runs for each subpool and select the highest-fidelity design. To evaluate the robustness and convergence of the optimization, we performed multiple independent design runs across diverse gene libraries and found consistent improvements in predicted fidelity (fig. S1).

The ability of OMEGA to design high-fidelity fragmentation sites depends on the properties of the target sequences. Sequences with high pairwise identity, elevated GC content, or repetitive elements could pose challenges because high sequence identity limits the availability of unique 4-bp sites and GC-rich sites can have lower fidelity than AT-rich sites (*20*). To evaluate these factors, we performed three separate computational analyses. First, to test the effect of sequence identity, we generated three sets of sequences representing identical (100%), high (~90%), and medium (~50%) pairwise sequence identities. For each case, we designed assemblies composed of either 4 or 12 fragments using 50 or 70 sites per subpool. Across all conditions, OMEGA consistently identified high-fidelity fragmentation site sets, indicating that sequence similarity has little impact on optimization performance (Fig. 1C). Even for identical sequences, different fragmentation patterns can be selected, allowing independent and accurate assembly reactions.

Next, to evaluate GC content, we generated synthetic gene sets with GC content ranging from 20 to 80%. OMEGA achieved high predicted fidelity across this range, with only a modest reduction at 80% GC content (Fig. 1D). Designs with 20% GC content showed no measurable loss in performance, consistent with previous reports that GC-rich overhangs exhibit a subtle downward trend in fidelity (*20*). However, OMEGA failed to find solutions for all three subpool replicates for sequences with 20 and 80% GC content, suggesting that low variability in nucleotide identity limits the number of GG site options. Last, to assess repetitive sequences, we designed assemblies for designed ankyrin repeat proteins (DARPins) containing five ankyrin repeats. OMEGA identified high-fidelity solutions regardless of pairwise sequence identity, demonstrating its ability to handle highly repetitive architectures. Collectively, these analyses show that OMEGA performs robustly across a wide spectrum of challenging sequence characteristics, including high sequence identity, extreme GC content, and internal repeats.

### Probing the limits of GG multiplexing for scalable gene assembly

OMEGA’s scalability depends on the degree of multiplexing within each subpool. Higher multiplexing allows more genes to be assembled per subpool and supports the assembly of longer genes composed of additional oligo fragments. The upper limits of multiplexing are determined by the number of unique GG sites that can be used without significantly reducing assembly fidelity. As the number of GG sites in a reaction increases, the risk of misligation between nonmatching sites rises, leading to a decline in assembly fidelity. The maximum number of GG sites that can be included in a single reaction while maintaining high efficiency and fidelity remains largely unknown, in part because GG assemblies are done under ideal conditions not reflected here. We designed several oligopool assemblies that mimic OMEGA conditions to systematically explore how set size and gene length affect both the size of an OMEGA library and the length of genes we can assemble.

**Fig. 2.**
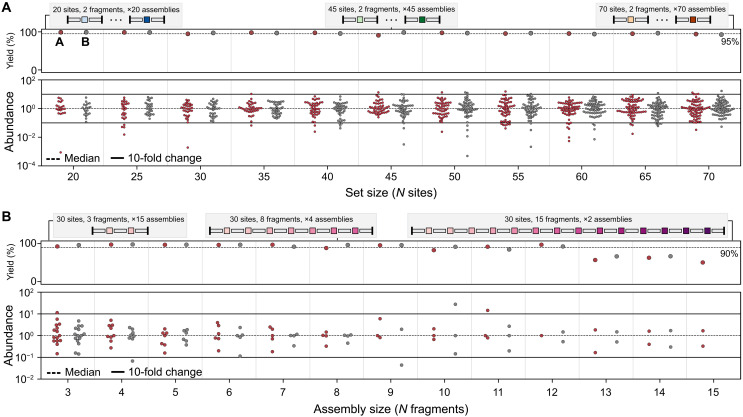
Fidelity of GG assemblies of varying complexities and sequence lengths. Target sequences consist of 25-mer fragments connected by a GG site assembled into a destination cassette. All trials were assembled using a high-fidelity GG protocol (details in Materials and Methods) and sequenced with more than 26,000× coverage. Number of GG sites indicates number of sites available for design—an additional two are used as vector ligation sites. Yield represents the percentage of NGS reads that are correct assemblies versus all reads. Abundance is represented as x-fold from the median abundant sequence. We consider sequences to be over- or underrepresented if they are more than 10-fold from the median. Designs inadvertently containing BsaI sites in the assembled construct were removed from analyses. (**A**) Fidelity of assemblies using 20 to 70 GG sites in increments of five. Red and gray dots represent two independent design trials. Most assembly sizes achieve >95% yield with a handful of underrepresented sequences. Sequences are rarely overrepresented. (**B**) Fidelity of assemblies with sequences using 3 to 15 fragments. Product yield remains constant around 90% until 13 fragments, at which point yield drops to ~65%. The distribution of sequences is typically within 10-fold of the median.

To assess the impact of multiplexing on assembly fidelity, we used OMEGA to design assemblies with varying numbers of GG sites per reaction. For each reaction, we assembled two 25-nucleotide oligomer (mer) barcodes into a linear destination cassette using a designed GG site between the two barcodes. The fully assembled construct was 112 bp in length, which contained the 62-bp barcode product. All barcode fragments were synthesized as 120–nucleotide (nt) oligos. We scaled the number of GG sites from 20 to 70 by adjusting the number of two-fragment constructs in the reaction. For example, we assembled 40 fragments to test a set of 20 GG sites and 140 fragments to test 70 sites. In addition to the designed GG ends, each fragment included a unique 25-mer sequence, allowing us to evaluate assembly fidelity using Illumina sequencing. Some of the 25-mer barcode sequences used in construct design inadvertently contained internal BsaI recognition sites. These sites caused the assembled constructs to be cleaved during GG assembly, resulting in severely reduced abundance in the final library (table S1). As a result, the effective library size—defined as the number of constructs without internal BsaI sites—was smaller than the total number of designs in some conditions. Unless otherwise noted, all analyses include only constructs that are free of BsaI sites in their assembled form. For each set size, we performed two assemblies using diverse sets of GG sites to evaluate variability between solutions (fig. S2). We then performed the OMEGA protocol by separately amplifying each subpool, performing GG assembly with a high-fidelity protocol. We analyzed fidelity, coverage, and bias using deep Illumina sequencing (Fig. 2A) for all assemblies.

Across all tested GG set sizes (20 to 70 sites), over 90% of sequenced assemblies were correct with most assemblies exhibiting >95% correct product. With the exception of one subpool, all target sequences were observed at least once. One 40-site subpool assembled 97% of target sequences. We assessed correct assemblies based on fragment order and did not consider sequence correctness. Thus, these errors reflect the impact of misligation events on assembly fidelity. The assembly products were highly uniform across set sizes, with 94% falling within 10-fold of the median-abundant sequence on average. While underrepresented sequences became more common as set size increased, overrepresented sequences remained rare across all conditions. Constructs that contained GG sites in the assembled gene were generally underrepresented, although still observed in most cases (fig. S3). We found that low-abundant constructs often used low-efficiency GG sites, had low fragment abundance after PCR, or a combination of both (figs. S4 and S5).

Next, we investigated how construct length affects OMEGA’s assembly fidelity by determining the maximum number of fragments that can be reliably assembled. Longer genes composed of more fragments will decrease assembly fidelity through increased partially assembled products due to the cumulative effect of multiple imperfect ligation events. We used OMEGA to design assemblies with constructs containing 3 to 15 fragments, ranging from 141 to 489 bp including the destination cassette. All fragments were synthesized as 120-nt oligos. Constructs with more fragments require more GG sites, making it difficult to distinguish the effects of multiplexing from construct length. To separate these factors, we designed subpools in which we held the total number of GG sites constant (30 construct sites and 2 backbone sites per reaction) while varying the fragment number per construct. This meant that reactions assembling shorter constructs contained more total constructs per reaction, whereas reactions assembling longer constructs contained fewer. For example, reactions assembling three-fragment constructs included 15 independent constructs, whereas reactions assembling 15-fragment constructs included only two constructs per reaction.

We used the OMEGA protocol to assemble designed constructs ranging from 3 to 15 fragments and analyzed assembly fidelity, coverage, and bias using Oxford Nanopore sequencing (Fig. 2B). For most assemblies using 3 to 12 fragments, over 90% of sequenced constructs were correctly assembled, and all target sequences were observed at least once. However, fidelity dropped sharply for constructs with more than 12 fragments. Assembly uniformity remained high up to eight fragments, with nearly all sequences falling within 10-fold of the median. Assessing assembly uniformity becomes challenging beyond eight fragments due to the low number of constructs in each reaction and may be more susceptible to variability in assembly efficiency. Some constructs inadvertently contained internal BsaI recognition sites within the assembled gene, which caused cleavage during assembly and resulted in these sequences being underrepresented in the final library (fig. S3 and table S2). We observed a similar trend in GG site efficiencies and fragment abundance in low-abundant constructs for shorter assemblies using fewer fragments (figs. S6 and S7).

OMEGA overcomes the traditional length-versus-scale trade-off in gene synthesis by combining the low per-base cost of oligopools with a simple, high-throughput assembly process, enabling construction of hundreds of user-specified genes. Because per-gene cost directly limits the number of constructs that can be built and tested, this approach greatly expands the scale of synthetic biology, metabolic engineering, and protein design. To compare costs, we modeled assemblies using 20 to 70 GG sites across gene lengths from 0.3 to 2.5 kb and compared them to Twist Bioscience fragment synthesis, which scales nearly linearly at 7 to 9 cents per base up to 5 kb. OMEGA’s pricing depends on library size, oligo utilization, and nonlinear oligopool costs (e.g., 2000 × 300-bp oligos cost 0.6 cents per base, whereas 6000 cost 0.3 cents). Across these conditions, OMEGA consistently lowered synthesis costs by at least 10-fold relative to individual fragment synthesis (fig. S8), demonstrating substantial economic advantages for large gene libraries.

### Scalable assembly of large gene libraries

We next sought to evaluate OMEGA’s ability to assemble large genetic constructs using the parameters established in our earlier experiments. To do this, we designed two gene libraries. The first contained 500 ribulose-1,5-bisphosphate carboxylase/oxygenase (RuBisCO) sequences with a median length of 475 amino acids (1425 nt), each assembled from seven fragments. The second library included 18 truncated Cas9 sequences comprising the first 866 amino acids (2598 nt), divided into 12 fragments. The full-length Cas9 gene is over 3 kb and too large to assemble with high fidelity (*23*). For both libraries, OMEGA was used to design oligopools with 300-nt oligos and up to 70 GG sites per subpool.

**Fig. 3.**
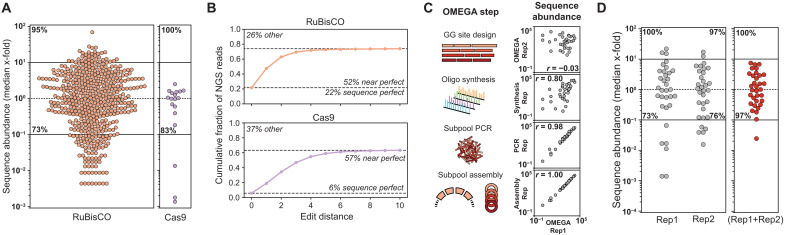
Assembly of RuBisCO and Cas9 libraries. All libraries were sequenced at least >3800× expected library size. (**A**) Sequence abundance of a RuBisCO and Cas9 library. Five hundred RuBisCO sequences were assembled in 46 pools using 70 GG sites (light orange). Eighteen partial Cas9 sequences were assembled in three pools using 70 GG sites (light purple). Sequence counts are combined from two replicate Cas9 libraries that use different GG site solutions. The number in the top of the plot indicates the percentage of sequences observed, while the lower number indicates the percentage of sequences within 10-fold of the median. (**B**) Error analysis for RuBisCO and Cas9 libraries. The cumulative fraction of PacBio sequence in reads at increasing edit distances (no. of mismatches/insertions/deletions) to the closest known library sequence. An edit distance of zero indicates sequence-perfect reads, edit distances of 1 to 10 may result from synthesis or sequencing errors, and edit distances beyond 10 likely come from assembly errors. (**C**) Steps in the OMEGA pipeline that may affect library bias. We used a test panel of 33 RuBisCO sequences and performed OMEGA replicates split at different points along the pipeline. A strong correlation in sequence abundance indicates that bias was introduced at an upstream experimental step. (**D**) Mitigating sequence bias by combining independent OMEGA replicates. Sequence abundance for two OMEGA replicates of the test panel of 33 RuBisCO sequences (gray) and the abundance for the combined library (orange). The number in the top of the plot indicates the percentage of sequences observed, while the lower number indicates the percentage of sequences within 10-fold of the median.

We assembled the 500 RuBisCO genes across 46 subpools and evaluated the resulting library using Pacific Biosciences (PacBio) sequencing to quantify the abundance of sequence-perfect constructs. Sequence-perfect constructs are reads that match the designed nucleotide sequence. We detected 95% of the designed sequences at least once, and 73% of all constructs were within 10-fold of the median abundance, indicating good library uniformity (Fig. 3A). We next assembled the 18 truncated Cas9 sequences in three subpools and performed duplicate assemblies using two distinct OMEGA design solutions. The two assemblies recovered 17/18 and 16/18 sequences, respectively, and their combined library covered all 18/18 targets (Fig. 3A and fig. S9). The merged Cas9 library exhibited strong uniformity, with 83% of constructs within 10-fold of the median abundance.

We next evaluated the sequence quality of the assembled RuBisCO and Cas9 libraries. For each PacBio read, we calculated the edit distance (total number of mismatches, insertions, and deletions) relative to its closest designed gene. The resulting distribution revealed three distinct categories (Fig. 3B and fig. S9): (i) sequence-perfect reads that were identical to the designed gene, (ii) near-perfect reads with 1 to 10 edits likely arising from oligo synthesis or sequencing errors, and (iii) reads with more than 10 edits indicative of assembly errors. For RuBisCO, 22% of reads were sequence-perfect, 52% were near-perfect, and 26% showed signs of assembly errors. For the merged Cas9 library, 6% of reads were sequence-perfect, 57% were near-perfect, and 37% likely contained assembly errors. These data suggest that synthesis-related errors occur roughly twice as often as assembly errors but are generally expected to be less disruptive to protein function. Improvements in oligonucleotide synthesis fidelity would further shift reads from the near-perfect to the sequence-perfect category.

Highly uniform gene libraries are critical for downstream screening applications. While the majority of the RuBisCO sequences were within 10-fold of the median, we sought to better understand the sources of bias in the OMEGA pipeline and identify opportunities to mitigate them. We identified four key stages that could introduce bias: (i) fragmentation site design, (ii) oligonucleotide synthesis, (iii) subpool PCR amplification, and (iv) the GG assembly reaction. To dissect the contributions of each step, we designed a controlled experiment using 33 RuBisCO sequences distributed across three subpools. We created a series of replicate libraries, each split at different branches of the pipeline (Fig. 3C and fig. S10). In the “design replicate,” two independent OMEGA design runs generated different fragmentation sites, and all downstream steps (synthesis, PCR, and assembly) were performed independently. The “synthesis replicate” had the same fragmentation site designs, but independent downstream oligo synthesis, subpool PCR, and assembly. The “PCR replicate” used the same oligos with independent PCR and assembly steps. Last, the “assembly replicate” used the same subpool PCR product with independent GG reactions.

All replicates exhibited sequence abundances spanning four orders of magnitude. As expected, replicates split later in the pipeline showed higher correlation. In particular, the PCR and assembly replicates were highly correlated, indicating that most sequence bias was already established before these steps. In contrast, design and synthesis replicates showed notably lower correlation, suggesting that these earlier stages, especially the fragmentation site design, contribute substantially to library bias. These findings point to a simple strategy for reducing bias: run multiple independent OMEGA design and assembly workflows and pool the results. Each run introduces distinct biases, which tend to cancel out when combined. We tested this approach with two independent OMEGA replicates of the 33 RuBisCO sequences. The individual replicates had 73 and 76% of their sequences within 10-fold of the median, whereas the combined library improved to 97%, substantially tightening the distribution (Fig. 3D and fig. S11).

### Large-scale assembly of natural and synthetic GFP genes

Fluorescent proteins (FPs) have diverse applications as reporters, affinity tags, tools for microscopy, and more (*24*). We have a rich repository of natural FP sequences and the capability to generate synthetic FPs (*9*, *25*), enabling the discovery of variants with improved spectral properties, brightness, photostability, and maturation times. However, large-scale exploration of FP sequence space remains limited by current gene synthesis capabilities because most sequences are more than 200 amino acids. To evaluate OMEGA’s performance, we tested its ability to synthesize hundreds of full-length FP genes with high accuracy.

**Fig. 4.**
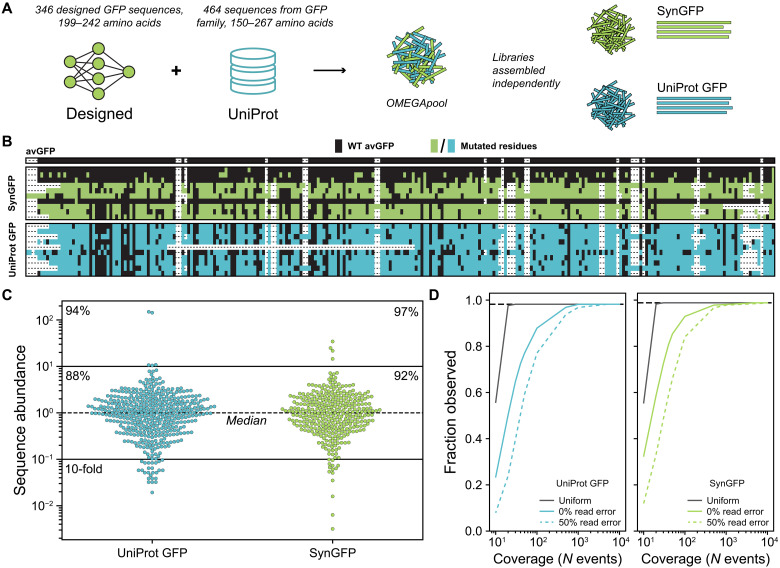
Assembly of 810 FP sequences using OMEGA. (**A**) FP sequences were either designed using machine learning (SynGFP) or curated from the UniProt GFP family (UniProt GFP). We used OMEGA to design an oligopool with both SynGFP and UniProt GFP sequences, which we assembled separately using a high-fidelity GG protocol. All subpools used less than 50 GG sites and encoded 2 to 24 constructs. (**B**) Sequence similarity for a random subset of 10 sequences from the SynGFP and UniProt libraries compared to wild-type (WT) avGFP. Mutated residues are with respect avGFP. Columns containing >95% gap characters were removed from the alignment. (**C**) Distribution of assembled SynGFP and UniProt GFP sequences. Sequence abundance is represented as x-fold from the median abundant sequence. Percentages within the 10-fold lines indicate fraction of library sequences within 10-fold of the median, and percentages in upper corners indicate fraction of sequences observed regardless of abundance. (**D**) Simulated sampling coverage to observe all library sequences. The probability to observe any individual sequence was calculated from the sequence distribution in (C), except for the uniform library, which assumes an equal probability to observe any given sequence. A 0% read error indicates that all reads and sequences map to a designed library sequence, whereas a 50% read error assumes that one in two reads will have some error that disqualifies that read. Error does not appear to significantly increase coverage burden.

We designed a panel of 810 FP genes, comprising both natural and synthetic sequences (Fig. 4A). We identified 464 GFP homologs from the UniProt GFP family (*26*). In addition, we generated a panel of 346 synthetic GFPs (SynGFP) using the generative protein language models (PLMs): CARP-640M (*27*), MIF-ST (*28*), and ESM-MSA (*29*). The SynGFP library consists of 316 sequences generated via Gibbs sampling, with the top 15 double mutants predicted by CARP and the top 15 double mutants predicted by MIF-ST (table S1). Details on the PLMs are provided in the next section. We assessed sequence diversity at the amino acid level using a multiple sequence alignment with *Aequorea victoria* green fluorescent protein (avGFP) and find that sequences vary significantly in both sequence identity and length (Fig. 4B). These sequences cannot be generated without custom synthesis and represent a challenging target for OMEGA.

We used the OMEGA software to design an oligopool for assembling our panel of 810 FP genes. All genes were first codon optimized for *Escherichia coli* using the GenScript codon optimizer; thus, even sequences with high sequence similarity at the amino acid level, such as the double mutants, range from 81 to 90% pairwise sequence identity at the nucleotide level (fig. S12). We distributed the 810 genes across 50 subpools regardless of nucleotide sequence, allowing each subpool to use up to 50 GG sites, two of which were fixed backbone sites AATG and TTAG. Each gene, ranging from 600 to 900 bp, was split into three or four fragments for assembly, and the median number of genes per subpool was 16. Notable outliers are a UniProt pool that contained two genes and a SynGFP pool that contained four genes. We followed a high-fidelity assembly protocol developed by Pryor *et al.* (*19*) to construct the FP library in 3 days with minimal hands-on time. First, we amplified each subpool by performing 50 PCR reactions in microtiter plates, followed by GG assembly within each subpool to construct individual genes. We then combined the subassembly products into separate UniProt-GFP and SynGFP libraries and completed a final column purification. We analyzed the assembled libraries using long-read PacBio sequencing at >1700× coverage to evaluate the assembly coverage, bias, fidelity, and error rates.

We analyzed the sequencing data to determine how many of the designed genes were present after OMEGA assembly (Fig. 4C). We only considered perfect sequence matches as correct assemblies. We found that 437/464 (94%) of the UniProt-GFP genes and 337/346 (97%) of the SynGPF genes were present at least once. We also found that the assembly protocol resulted in uniform gene abundance, with 88% of the UniProt-GFP genes and 92% of the SynGPF genes falling within 10-fold of the median-abundant sequence. Biases in sequence abundance reduce screening efficiency by requiring library oversampling to detect low-abundance sequences. We estimated the screening coverage needed to observe each sequence at least 10 times. We compared our assembled OMEGA libraries to an idealized scenario where all sequences are present in equal abundance (Fig. 4D). We found that our libraries generally needed 100× more screening to achieve the same coverage as a uniform library. We also found that simulated libraries with 50% incorrect assemblies only moderately decreased screening efficiency. 

The UniProt-GFP and SynGFP libraries contained overrepresented sequences, prompting us to investigate their cause. While most subpools contained 16 genes, we found that the overrepresented genes originated from remainder subpools with only two genes in the UniProt-GFP library and four genes in the SynGFP library. This likely occurred because our protocol did not normalize the output from each subpool, and smaller assemblies are more efficient. Biases in sequence abundance were largely attributed to uneven sequence distributions across subpools. To address this, we reassembled the SynGFP library while excluding these remainder subpools, which improved gene uniformity and eliminated overrepresented sequences (figs. S13 and S14).

The final OMEGA-assembled genes likely contain errors originating from the starting oligopool (*30*). With a fixed per-base error rate, longer genes are more likely to contain errors, and this places an upper limit on the length of error-free genes (Fig. 3B) (*23*). Even when fragments assemble correctly, errors from the original oligos can persist. To estimate the fraction of error-free genes, we mapped OMEGA library reads to the target reference sequences and found that over 60% of correctly assembled sequences were error-free (fig. S15). This number is consistent with Twist Bioscience’s reported per-base error rate of 1:3000. In addition, a separate analysis from our GG multiplexing experiments above showed that synthesis errors rarely occurred in GG or BsaI sites, suggesting that synthesis errors do not significantly affect assembly fidelity (fig. S16).

### OMEGA accelerates generative design and screening of synthetic FPs

Generative protein design models can generate vast numbers of sequences with targeted structural and functional properties. Their ability to sample the high-dimensional sequence landscape creates diverse designs with the potential to uncover new and useful proteins (*31*, *32*). However, bringing these designs to life remains a bottleneck, as synthesizing individual genes is costly, especially for larger proteins. Scalable gene synthesis methods would bridge this gap in the design-synthesis-testing pipeline to accelerate protein discovery.

We tested OMEGA’s ability to assemble genes encoding FPs generated by PLMs. PLMs learn information-rich protein sequence representations and can be used to direct the generation of new sequences through masked language modeling or next token prediction. We generated GFP variants using three different PLM methods. The first was CARP-640M (*27*), which is a masked language model of protein sequences. The second model was MIF-ST (*28*), which is a structure-conditioned masked protein language model. The final model was ESM-MSA (*29*), a transformer-based PLM that generates novel protein sequences through masked language modeling from a multiple sequence alignment input.

We generated 100 sequences with CARP-640M, 100 sequences with MIF-ST, and 116 sequences with ESM-MSA using Gibbs sampling. These sequences were 80 to 95% identical to the nearest natural GFP. We also used the MIF-ST and CARP-640M models to predict the top 15 avGFP double mutants, defining a population of 30 designs with high sequence similarity at the nucleotide level distributed throughout assembly pools (fig. S11). As described in the previous section, we applied OMEGA to assemble these 346 SynGFP genes and found that 337/346 (97%) were correctly assembled (Fig. 4C and fig. S13). This demonstrates OMEGA’s ability to accurately assemble genes with high sequence similarity, highlighting its effectiveness for constructing both diverse and closely related variants.

**Fig. 5.**
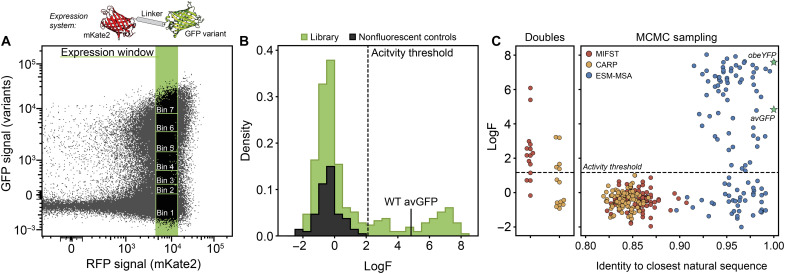
Characterization of SynGFP library using FACS. (**A**) Library sequences were expressed as a fusion protein with mKate2, which was kept constant between library variants and indicates level of expression. Cells were sorted into seven bins of GFP fluorescence approximately uniformly distributed across a log-scale within a narrow mKate2 window to control for expression. (**B**) We calculated a LogF for each sequence averaged from two replicates. We calculated an activity threshold by fitting observed library sequences with a mixture model to determine the intersection of functional and nonfunctional populations. We also calculate the LogF for an additional 76 incorrect assemblies that serve as nonfluorescent controls. (**C**) GFP fluorescence plotted by model and sequence identity. Double mutants are two mutations from avGFP. MCMC sampling sequences are plotted by pairwise identity to the closest natural sequence in the UniProt GFP family. Two known FPs are included as references and indicated by green stars.

We evaluated the fluorescence of the assembled SynGFP variants using fluorescence-activated cell sorting (FACS) to sort them into bins based on increasing fluorescence levels (Fig. 5A and table S3). To estimate relative brightness, each GFP variant was expressed as a fusion with a constant mKate2 reference protein, and a log fluorescence (LogF) value was calculated based on the variants’ distributions between bins. The screen also included wild-type avGFP as a positive control and incorrectly assembled genes as assumed nonfluorescent negative controls. We fit a Gaussian mixture model to categorize sequences as fluorescent/nonfluorescent (fig. S17) and found that 26% of all library designs exhibited fluorescence (Fig. 5B). We then evaluated each model’s SynGFP design performance (Fig. 5C). We found that the MIF-ST model successfully designed functional double mutants 80% of the time and was able to identify two variants with a relative brightness higher than wild-type avGFP. The CARP model was less successful with only 35% functional double-mutant designs. Using more aggressive Markov chain Monte Carlo (MCMC) sampling to generate more sequence diversity, both MIF-ST and CARP models fail to design functional FPs. We found that MCMC sampling of the ESM-MSA model introduced less mutations overall and 61% of generated designs were fluorescent. Two of the sequences designed by ESM-MSA shared 100% sequence identity with known FPs *Obelia sp.* yellow fluorescent protein (obeYFP) and an uncharacterized cyan fluorescent protein.

## DISCUSSION

Protein engineering relies on large sequence libraries to broadly sample sequence-function space and discover new proteins (*33*, *34*), but current gene synthesis approaches limit the scale of this exploration. To overcome this, we developed OMEGA, a simple, low-cost, and efficient method for assembling oligopools into large, diverse gene libraries. OMEGA uses a computational pipeline to split genes into short fragments that can be ordered as oligopools and assembles them using a hierarchical GG cloning strategy using DAD. We demonstrated that OMEGA can reliably assemble constructs of up to 12 fragments and can use up to 70 GG sites while reducing synthesis costs by more than 10-fold. To validate the method, we assembled a library of 810 natural and engineered GFP sequences and recovered over 94% of the target sequences. Overall, OMEGA offers a scalable, cost-effective, and robust solution for gene library assembly with broad applications in protein engineering and synthetic biology.

OMEGA leverages standard molecular biology techniques to achieve high-fidelity assembly of large gene libraries through GG multiplexing. GG cloning is well established, with commercially available reagents that enable robust and reliable assembly. Notably, assembling a single OMEGA subpool follows the same straightforward workflow as clonal gene assembly using GG cloning. DropSynth, another high-throughput gene assembly method, scales PCA by using microemulsions to encapsulate custom-barcoded beads, each carrying the genetic components for individual genes (*16*). This approach requires a complex, multistep process, including the preparation of custom-barcoded beads and the precise optimization of 20-bp DNA overlaps for each fragment.

OMEGA improves gene recovery compared to existing high-throughput gene assembly methods. DropSynth was used to assemble 346 dihydrofolate reductase (DHFR) genes in duplicate assemblies using different codon sequences. Individual libraries observed 87 and 62% of genes (92% across both assemblies) (*35*). In a larger assembly targeting 1536 DHFR constructs, 68 and 59% of genes were observed in two assemblies (~80% overall). In contrast, OMEGA observes 94 to 97% of all target sequences in a single assembly with libraries of similar or larger size. The SynGFP and UniProt GFP can be considered as a single large library since all sequences were ordered in a single oligopool and assembled in the same way. In total, we observe 774/810 genes (95%) of all GFP constructs, suggesting that library coverage does not significantly decrease with library size. Furthermore, OMEGA maintains high fidelity for constructs with up to 12 fragments while DropSynth assembly fidelity significantly drops around five fragments. We expect that the easy integration of OMEGA assemblies into standard GG cloning workflows, combined with our high fraction of observed target sequences, will enable scientists to construct gene libraries with ease.

Innovation in synthetic biology is driven by our capacity to design and synthesize custom DNA. GG cloning is the foundation of many cloning workflows to efficiently design DNA sequences using multifragment assemblies. DAD uses experimentally derived overhang annealing and ligation preferences to identify high-fidelity GG cloning junctions, significantly expanding the scope of GG cloning (*19*). Here, we extend the complexity of DAD assemblies from 52 GG sites to 70 in a single reaction, using ~30% of all possible 4-bp overhangs and achieving ~95% assembly fidelity. We also demonstrate that assemblies with up to 12 fragments maintain high fidelity, underscoring DAD’s potential for constructing even larger, high-complexity assemblies beyond protein-coding genes. The success of our assemblies, which each use unique combinations of GG sites, emphasizes that DAD is a robust method to design novel sets of GG sites that yield high-fidelity assemblies, reducing constraints based on sequence and assembly size.

Most assembly products fall within 10-fold of the median abundant sequence in an OMEGA library, but some sequences fall above or below this range. We investigated different sources of this bias in the OMEGA pipeline and found that the identity of the GG sites was the most significant factor affecting assembly product abundance. We also observe that libraries assembled using 70 GG sites exhibit a higher frequency of overrepresented products than libraries using fewer GG sites. Combining the assembly products of replicate OMEGA assemblies using different GG sites seems to reduce the bias and increase the overall library coverage. Thus, depending on library size and applications, one should balance the trade-offs of assembly complexity and labor versus library bias. In some cases, it may be useful to perform replicate subpool assemblies using different GG sites, especially if the end goal is to isolate clonal genes.

OMEGA relies on input oligo pools that are created through solid-phase DNA synthesis. OMEGA is inherently limited by the baseline error rate of DNA synthesis, which introduces occasional nucleotide substitutions, insertions, or deletions into the oligo pool. These errors accumulate with increasing oligo length, and they cannot be entirely eliminated with current technologies. Nevertheless, OMEGA is able to assemble a high fraction of sequence-perfect constructs, even for long genes. Furthermore, a subset of synthesis-derived nucleotide errors result in silent mutations that do not alter the encoded protein sequence. In many cases, proteins can also tolerate a small number of random mutations without losing function, meaning that imperfect constructs can still contribute valuable diversity to downstream screening. Existing error correction methods such as enzymatic error correction or multiplex oligonucleotide library purification by synthesis and selection may help to further reduce errors (*30*, *36*).

While solid-phase synthesis imposes a fundamental ceiling on oligo fidelity, it is a rapidly improving technology. As synthesis quality advances, we expect OMEGA’s capabilities to improve in parallel. Higher-fidelity and longer oligos will naturally extend the length and accuracy of assemblies. For instance, recent advances such as Twist Bioscience’s 500-bp multiplexed fragments will theoretically enable OMEGA to assemble constructs up to 5 kb. Continued improvements in DNA synthesis will expand the practical limits of OMEGA, enabling multikilobase assemblies and more accurate libraries.

OMEGA can reduce the per-gene synthesis cost by ~10-fold compared to clonal gene synthesis, but it generates pooled libraries that must be screened to identify functional variants. Because these libraries are pooled and exhibit sequence bias, we estimate that 20- to 100-fold oversampling is needed to capture the full diversity of the library. The cost-benefit of this trade-off depends on the protein system and the availability of medium- or high-throughput screening methods. OMEGA is less practical in contexts where screening throughput is limited, such as those requiring protein purification or detailed analytical measurements like liquid chromatography–mass spectrometry. Nonetheless, when screening is feasible, OMEGA provides a scalable, cost-effective solution for building large and diverse gene libraries.

We are in an information age of biological data where it is increasingly important to target sequence space with precision-designed libraries to develop and refine machine learning (ML) engineering strategies. OMEGA is a simple gene assembly method that excels in assembling libraries of sequence-diverse constructs with high coverage from oligopools. With GG cloning, OMEGA libraries integrate seamlessly into established cloning workflows and mitigate financial barriers that limit the scope of custom libraries. We envision that OMEGA will empower data-driven engineering with its simple and affordable library workflow.

## MATERIALS AND METHODS

### Implementation of OMEGA software

The OMEGA software automates library design to assemble diverse genetic constructs from oligopools. Users provide a list of codon-optimized sequences and OMEGA returns two files detailing the precise oligo sequences to order and a list detailing the contents of each subpool. Detailed pseudocode is given in the Supplementary Materials. The main function of the OMEGA software is to use DAD to find fragmentation patterns with optimal GG sites to enable high-fidelity assembly of individual genes. We calculate the fidelity as by Pryor *et al.*. (*19*) that assumes each GG site will be used in a single sequential assembly. The overall fidelity is thus the product of the individual site’s predicted fidelity, which estimates the likelihood of a correct ligation in the presence of other GG sites in the set.

To start, the user provides a list of codon-optimized sequences and specifies the Type IIS restriction enzyme and the maximum number of GG sites that should be used for library assembly (GG site budget). If needed, the user may also provide two backbone ligation sites that are included in the fidelity prediction and set size calculation. OMEGA begins by estimating the number of fragments needed to encode the longest gene in the library and uses this value as the target number of fragments to break all genes into. Breaking genes into the same number of fragments reduces potential library bias from shorter genes that are more efficiently assembled than longer genes. OMEGA calculates how many pools are required to encode all genes based on gene length and GG site budget and uniformly distributes genes among the pools such that the pool with the most genes has, at maximum, one more gene than the pool with the fewest genes.

Next, OMEGA computationally designs fragments for each subpool by optimizing the fidelity of the GG sites created by different fragmentation patterns. OMEGA initializes pools by breaking genes into fragments of equal length. Any sites that are duplicated are shifted left or right by 1 bp until all starting sites are unique within a subpool. The challenge of fragment design can be posed as a combinatorial optimization problem guided by the predicted fidelity of the current set of GG sites. Each gene can be broken into one of several legal fragmentation patterns that yield different sets of GG sites. This problem space is too large to solve absolutely—for example, a pool with 10 genes that each have three break points and each break point has 25 GG site options has ~10^41^ site combinations (including combinations with repeated GG sites).

We apply SA to approximate an optimal solution from these potential options. For every optimization step, we shuffle a random GG site to propose a new set of GG sites that differ by one position. If the predicted fidelity is better than the previous solution, the new site is accepted. If the solution is worse, then it is accepted with probability $e^{∆\text{Fidelity}/T}$ where *T* is decreased along a logarithmic temperature gradient ranging from 5 × 10^−3^ to 10^−5^ over 2000 to 10,000 optimization steps. This optimization process is completed *N* number of times using different random seeds to find different solutions. The best of *N* optimization runs is selected for subpool design. More optimization steps and optimization runs are recommended for assemblies using more GG sites. OMEGA automatically packages genes across all subpools into a list of fragmented sequences ready to be ordered with all required restriction enzyme sites and amplification primers added in. All oligo fragments are padded with random DNA sequences between the BsaI binding site and primer binding site to make oligos a uniform length. An additional list is provided that provides details on each pool, including library genes, predicted fidelity, GG sites, primers for pool amplification, and the random seed used to design the gene fragments.

The code to run OMEGA is on GitHub and archived with Zenodo. Our code can be run locally with the provided environment or using a Google Colab notebook. We use jsonargparse to customize OMEGA’s runtime parameters to suit specific use cases. For example, users may change the restriction enzyme, number of GG sites used in each subpool, backbone sites, and more. We provide an example configuration file with all parameters users can change in the Supplementary Materials and provide examples of library optimizations in the code published with this paper.

### OMEGA simulations

We performed a series of simulations to test how sequence identity affects overall fidelity. For each simulation, sequence pools were either identical with an average pairwise identity of 100%, high identity with 86 to 90% average pairwise identity, or medium identity with 49 to 50% average pairwise identity. Each simulation was performed in triplicate.

First, we designed pools of 4- and 12-fragment sequences based on the full avGFP sequence (UniProt: P42212) and the first 865 amino acids of Cas9 (UniProt: J7RUA5), respectively. High-identity four-fragment sequences are independent optimizations of the avGFP sequence using the GenScript codon optimization tool and *E. coli* as the host organism. To generate high-identity 12-fragment sequences, we first designed a template sequence for the truncated Cas9 sequence using a set of codons optimized for folding in *E. coli* (*37*). Next, we introduced synonymous mutations to the template until the desired diversity was achieved. To generate 4- and 12-fragment medium-identity sequences, we randomly selected a representative codon sequence and introduced random amino acid mutations until the desired diversity was achieved. For each simulation, three random sequences were selected for the identical sequence pools. This process was used to design pools with 50 GG sites and 70 GG sites.

The second set of simulations varies the GC content of target sequences from 20 to 80% in increments of 10%. First, we generated three random nucleotide sequences for each GC content that were 714 bp in length for four-fragment sequences and 2595 bp in length for 12-fragment sequences. These serve as the sequences used in the identical sequence pools and templates to generate high- and medium-identity sequences. Next, we introduced random mutations to the template sequences until the desired sequence diversity was achieved. The actual GC content of mutated sequences was kept within 1% of the target GC content.

The third set of simulations uses DARPin as a model for repeating amino acid motifs. First, we used the GenScript codon optimizer to optimize the DARPin sequence [Protein Data Bank (PDB): 2QYJ] for each high-identity sequence. We selected three high-identity sequences as template sequences to generate medium-identity sequences. We introduced random amino acid mutations until the desired diversity was achieved. Three high-identity sequences were selected as random sequences for identical sequence pools.

The number of sequences used in each pool is as follows: four-fragment sequence pools had 16 sequences using 50 GG sites and 22 sequences using 70 GG sites, 12-fragment sequence pools had four sequences using 50 GG sites and six sequences using 70 GG sites, and three-fragment sequence pools had 34 sequences using 70 GG sites. All pools were optimized using AATG and TTAG as backbone sites. Pools using 50 GG sites were optimized 25 times for 3000 steps and pools using 70 GG sites were optimized 50 times using 5000 steps.

### Design of diverse GG sets

We used OMEGA to design high-fidelity, diverse GG sets containing between 20 and 70 sites in increments of five. Each set also contained an additional two vector ligation sites CTAA and CATT. Thus, the total number of GG sites in a set size of 70 is 72 including the vector sites, but only 70 is used for sequence design. No restrictions were placed on GG site options except for keeping vector ligation sites fixed. We designed 50 sets for each size and identified the two most diverse sets using pairwise Hamming distance as a diversity metric for a total of 22 unique GG sets (11 sizes, two replicates). Sets are referred to by their size and replicate (e.g., sets 30a and 30b refer to our two diverse sets containing 30 sites). All 22 sets are used in the two-fragment assemblies that vary GG site number. The assemblies testing the effect of fragment number use sets 30a and 30b for all fragment numbers.

### Design of OMEGA parameterization assembly sequences

We designed synthetic sequences for assemblies varying GG site number and assemblies varying fragment number used to parameterize OMEGA. The synthetic sequences consist of a series of unique 25-mer barcodes joined by a variable GG site that is not part of the barcode sequence. Barcode sequences were chosen from a set of super orthogonal barcode sequences designed by Xu *et al.* (*38*). All barcodes vary by at least 15 bp to allow for good discrimination of sequences using both Illumina and Oxford Nanopore sequencing platforms. Some barcode sequences inadvertently contained BsaI recognition sites, causing those constructs to be digested during assembly and resulting in very low abundance. These erroneous assemblies were excluded from subsequent analyses and are marked with X in the relevant figures.

The first and last fragments are flanked by the upstream and downstream vector ligation sites CTAA and CATT.

For each of the 22 unique sets of GG sites, we designed two-fragment assemblies using the above synthetic sequence design scheme. For the assemblies where we varied the number of fragments, we use sets 30a and 30b to randomly assign the GG sites connecting 3 to 15 fragment assemblies. For each fragment number, we designed as many sequences as possible given a fixed GG site budget of 30.

To design the genes as oligos, we fragmented sequences by barcode and added corresponding upstream and downstream GG sites, BsaI restriction sites, and primer binding sites. We added equal padding to the left and right of the barcode between the BsaI restriction site and primer binding site to ensure that all sequences were 120 nt. A list of sequences, GG sites, and primers is archived with Zenodo (DOI: 10.5281/zenodo.17637645).

### RuBisCO library design and assembly

We selected 500 RuBisCO sequences from the Swiss-Prot “RuBisCO large chain family” (accessed 23 May 2025) that were between 440 and 490 amino acids and did not contain any ambiguous amino acid codes. We used OMEGA to design high-fidelity GG sites using up to 70 GG sites and used AATG and TTAG as fixed vector ligation sites. Each pool was optimized 50 times for 5000 steps. Individual fragments were ordered as 300-nt oligos. Sequences shorter than 300 nt were randomly padded to 300 nt, ensuring that the padding did not contain BsaI sites. The full library consisted of 46 assembly pools with a median of 11 sequences per pool.

We selected 33 sequences from the RuBisCO library corresponding to three subpools to design a series of mini libraries that serve as controls for different aspects of the OMEGA assembly pipeline. We designed an initial mini library, called OMEGA Rep1, using OMEGA as described above. The three subpools we selected were present in the full RuBisCO library using a different set of GG sites for gene assembly; this served as a technical replicate for OMEGA, called OMEGA Rep2. For the synthesis control, we ordered a second set of the oligos used in the OMEGA Rep1 assembly indexed by different unique primers and used these as input to the assembly. For the PCR control, the OMEGA Rep 1 subpools were amplified a second time and used in the assembly. For the GG assembly technical replicate, the same PCR product used to assemble OMEGA Rep 1 was used in a second GG assembly. For all mini libraries, the protein codon sequence was fixed, although the random padding was variable except between OMEGA Rep1 and the synthesis control. All sequences and primers are archived with Zenodo (DOI: 10.5281/zenodo.17637645).

### Cas9 sequence design and assembly

We selected 18 sequences from the CRISPR-associated endonuclease Cas9 family on UniProt (accessed 23 May 2025) that were between 1048 and 1053 amino acids. Sequences were shortened to the first 866 amino acids of the full-length sequence. We used OMEGA to design high-fidelity GG sites using up to 70 GG sites and AATG and TTAG as vector legation sites. Subpools were optimized 50 times for 5000 steps. Each subpool contained six sequences for a total of three subpools. We designed GG sites in duplicate for each subpool, resulting in replicate libraries that contained identical sequences and used different GG sites for assembly. Both libraries were ordered as 300-nt oligos with padding added to ensure that all oligos were equal length.

### Next-generation sequencing and analysis for RuBisCO and Cas9 libraries

We pooled the amplified libraries and applied PacBio HiFi sequencing. We first demultiplexed pooled samples by their unique barcodes, which each correspond to a specific library. We matched barcodes to the first and last 20 bases for a given read, allowing up to five insertions in the leading and trailing bases to account for sequencing errors common at the ends of reads. Any reads that did not begin and end with a valid pair of barcodes were removed from subsequent analyses. We aligned sequences using the pbmm2 align program with HiFi read presets and removed any read with a quality score of <30 in the aligned region. The number of reads remaining after this step determined the total number of next-generation sequencing (NGS) reads for each library.

For all sequence abundance and library distribution analyses, the number of sequence-perfect matches was counted for each library sequence. A sequence-perfect match is a perfect match at the nucleotide level and does not allow mismatches, insertions, or deletions. A sequence is considered observed if it has at least one count. For individual libraries, sequence counts were divided by the median abundant sequence. For analyses combining counts from more than one library, sequence counts were normalized by the total number of sequence-perfect reads, summed, and divided by the median abundant sequence.

We calculated the edit distance from each read to the closest related library sequence for all reads that mapped with full coverage to a library sequence. Edit distance is defined as the number of mismatches, insertions, or deletions required to make two sequences equal to one another. We considered a mapped read full coverage if the length of the aligned sequence was within 10 nt of a library sequence. For individual libraries, the frequency for each edit distance was divided by total NGS reads and reported as a cumulative sum. For combined libraries, edit distance frequency was first normalized to total NGS reads, summed, and then divided by the normalized total NGS reads. All libraries were sequenced at least >2900× after read quality filtering.

### GFP sequence design

For the SynGFP library, we designed 346 GFP sequences using different ML models. First, we used MIF-ST and CARP-640M to score every possible double mutant to wild-type GFP and selected the top 10 from each model. Next, we performed Gibbs sampling using MIF-ST, CARP-640M, and ESM-MSA, as described, to diversify natural GFP sequences (*39*, *40*). Beginning with a natural sequence, at each iteration, 10% of positions were masked, and the model was used to predict the probability of each amino acid at each masked position. For each of 10 burn-in steps, the masked positions were sampled from the predicted distributions. Then, for each of 10 sampling steps, each masked position was set to its most probable amino acid, resulting in 10 generated sequences per starting natural sequence. For CARP-640M, 10 starting sequences were randomly chosen from a set of putative GFP homologs. For MIF-ST, samples used the PDB 2WUR structure and corresponding sequence. For ESM-MSA, 10 subsets of 63 aligned homologs and one starting sequence were randomly chosen as starting points. This results in 100 generations for each model.

For the UniProt library, we randomly selected 464 sequences ranging between 200 and 250 amino acids from the UniProt GFP family (accessed 19 July 2022). All sequences were codon-optimized for yeast using the GenSmart codon optimization tool from GenScript and specified that optimized sequences excluded BsaI sites. We used OMEGA to design the SynGFP and UniProt GFP libraries separately, although the same design parameters were used. In both cases, we specified that no more than 50 GG sites could be used in each subpool and that each pool must include AATG and TTAG as vector ligation sites. Subassembly pool sizes ranged from 20 to 4 genes for the SynGFP library and 24 to 2 genes for the UniProt library. All genes were broken into either three or four fragments. We ordered the OMEGA library as 300-bp oligos and added random DNA padding between the primer amplification site and BsaI restriction site to ensure that all oligos were 300 bp. Subassembly pools are indexed by a unique combination of orthogonal primers for selective amplification (*22*). All primers and sequences are archived with Zenodo (DOI: 10.5281/zenodo.17637645).

### High-fidelity GG assemblies

All oligopools were resuspended to a concentration of 2 ng/μl using nuclease-free water. KAPA HiFi HotStart ReadyMix 2× (Roche, no. KK2601) was used for all PCRs. Zymogen DNA Clean and Concentrate (Zymo Research, no. D4006) was used for all cleanups. All DNA was eluted using the Zymogen Elution Buffer heated to 65°C to improve DNA yield. For subpool PCRs, 1 ng of template DNA was amplified using forward and reverse primers at a final concentration of 0.3 μM and a total reaction volume of 25 μl with the following PCR protocol: 98°C, 3 min ➔ (98°C, 15 to 20 s ➔ 61°C, 15 s ➔ 72°C, 15 s) × 35 cycles ➔ 72°C, 1 min. PCR products were cleaned up and normalized to 50 or 20 ng/μl.

All GG assemblies follow a high-fidelity protocol adapted from Potapov *et al.* (*20*). Briefly, insert and vector are digested together with BsaI–HFv2 (New England Biosciences, no. R3733L) for 2 hours at 37°C (15 U BsaI, 1× T4 Ligase Buffer, 20 μl reaction volume). An 18:1 molar ratio of insert to vector was used to assemble the SynGFP and UniProt GFP libraries, and a 5:1 molar ratio of insert to cassette was used for all other assemblies. After 2 hours, 1000 U of T4 Ligase (New England Biosciences, no. M0202M) was added to each assembly and further incubated for 18 hours at 37°C, followed by heat inactivation (65°C, 20 min). Subpools were pooled by library and cleaned up. Some assemblies were further enriched using PCR using the following protocol: 98°C, 3 min ➔ (98°C, 15 to 20 s ➔ 61°C, 15 s ➔ 72°C, 45 s/kb) × 16 cycles ➔ 72°C, 1 min/kb. Exact extension times vary by product length and are included with the specific assemblies below. PCR products were digested with BsaI–HFv2 for 30 min at 37°C (15 U, 1× CutSmart buffer) and cleaned up.

Sequences from the OMEGA parameterization assemblies varying GG set size and fragment number were assembled into a linear destination cassette that we designed and ordered as duplexed DNA from IDT. We designed reverse-oriented BsaI binding sites separated by five nucleotides using CTAA for the upstream site and CATT for the downstream site. There is a randomized 10-bp region upstream of the first BsaI site. Primers flank both GG sites for subsequent PCR after assembly. The GG product from each of these assemblies was amplified using extension times of 15 s per cycle and 1 min final extension (primers: pr.subra_84 and pr.subra_88) and cleaned up before sequencing.

The RuBisCO library, controls, and Cas9 libraries were assembled into linear destination cassettes using a 5:1 molar ratio of insert to cassette. Each cassette contained reverse-oriented BsaI sites separated by five nucleotides and flanked by unique pairs of primers used to barcode the assemblies. Cassettes were ordered from IDT as duplex DNA. Assembly targets were enriched from the GG product using PCR with the following extension times: RuBisCO sequences used 1 min 8 s per cycle and 1 min 30 s final extension, and Cas9 sequences used 1 min 57 s per cycle and 2 min 36 s final extension. For Cas9 assemblies, we gel-extracted (1.3% agarose gel) the target assemblies from the amplified GG product using a Zymogen Gel Recovery Kit (Zymo Research, no. D4001) and repeated the PCR enrichment on the extracted product.

The UniProt and SynGFP libraries were assembled into a modified pqe30 expression vector previously described (*5*). We started from a related backbone (Addgene, no. 74748) (*41*), which expresses a KillerOrange-GFP fusion protein. We replaced KillerOrange with an mKate2 construct ordered from Twist Bioscience using the Bam HI and Not I restriction sites. We next replaced GFP with the BsaI sites AATG and TTAG using PCR and blunt-end ligation (pr.001 and pr.002). We also created a version without mKate2 using PCR to remove the mKate2 sequence followed by blunt end ligation (primers: pr.003 and pr.004). We assembled the SynGFP library into the vector with mKate2 and UniProt GFP library into the vector without mKate2. We also performed a second assembly of the SynGFP sequences called SynGFP B, which excludes two pools from the SynGFP assembly that included three-fragment genes or had fewer genes than other subpools. A list of all subpools and sequences used in each assembly is archived with Zenodo (DOI: 10.5281/zenodo.17637645).

### NGS processing for parameterization assemblies using variable GG sites

We applied 2 × 150 Illumina sequencing to all GG assemblies. This 2 × 150 sequencing can read sequences up to ~300 bp. Correct two-fragment assemblies are 62 bp, but assemblies with up to eight fragments are short enough for 2 × 150 reads. We expect that 2 × 150 sequencing can adequately detect assembly results. All subpools were sequenced with a minimum of 26,000× coverage. The full data processing pipeline is included in the Supplementary Materials. Briefly, we merged forward and reverse reads, trimmed extraneous DNA outside of the insert region (30 bp from start, 20 bp from end), and discarded reads with a predicted error rate of >1 bp. We then mapped individual barcode sequences against reads to define an assembly code, which is determined by the order in which the barcodes were assembled and identifies assemblies independent of sequence. Because oligopools contain synthesis errors and our objective is to assess the effect of GG set size on assembly fidelity, we allow up to three mismatches in the barcode sequence. We discarded any partially matched reads, any assembly that contained barcodes from more than one subpool, and if the trimmed read did not start and end with the expected flanking GG sites.

We use the assembly code to differentiate correct and incorrect assemblies. All counts were aggregated by assembly code to assess GG site fidelity independent of synthesis errors in the barcode sequence. Fully mapped reads define the full read population, and correct assemblies are those with predicted assembly codes. Target sequences were considered assembled if the corresponding assembly code was observed at least once. Yield is calculated by the number of correct reads divided by the number of fully mapped reads for each population. When indicated, constructs with BsaI in the assembled product are excluded when calculating fold change from median abundant sequence and library coverage. All populations were sequenced with at least 26,000× coverage after read filtering.

### NGS processing for parameterization assemblies using variable fragment numbers

We applied Oxford Nanopore Technologies (ONT) Sequencing to all GG assemblies. The expected size of assemblies is between 141 and 489 bp, and some incorrect assemblies may be longer. ONT sequencing is less biased by variable length sequencing input and can sequence longer constructs than Illumina. All subpools were sequenced with a minimum of 35,000× coverage. The full data processing pipeline is included in the Supplementary Materials. Briefly, we used usearch to map individual barcode sequences and flanking primer sequences to ONT reads, allowing for up to three mismatches. We kept reads that satisfied the following conditions: (i) Both upstream and downstream primers were present once and in the same direction, (ii) aligned barcodes covered the whole insert region, and (iii) all barcodes were from a single assembly pool. For all analyses, we considered the total number of reads as those with full-coverage alignments and barcodes from the same assembly pool. We used the order of barcode assembly to assign each read an assembly code and consolidated counts by assembly code. Correct reads are those with predicted barcodes. Target sequences were considered assembled if the corresponding assembly code was observed at least once. Yield is calculated by the number of correct reads divided by the number of fully mapped reads for each population. When indicated, constructs with BsaI in the assembled product are excluded when calculating fold change from median abundant sequence and library coverage. All populations were sequenced with at least 35,000× coverage after read filtering.

### Sequencing for oligopool

To assess the quality of amplified oligopools, we applied 2 × 150 Illumina sequencing to the amplified oligos for each subpool used in the OMEGA parameterization assemblies. To process the data, we used the 32-bit version of usearch (*42*). We include the full data processing pipeline in the Supplementary Materials. Briefly, we merged forward and reverse reads and filtered out any reads with a predicted error rate >1 base. We counted the frequency of each unique sequence and discarded any sequence with <10 counts.

We generated two alignments from the oligopool data. To assess synthesis errors across the full oligo sequence, we used the “-search_oligodb” command to map the designed oligo sequence to Illumina reads and allowed up to three mismatches. Only reads that were 120 bp were kept. Correct sequences were those with perfect matches to designed oligos, and incorrect sequences contained at least one error. To assess synthesis errors in the BsaI binding site and GG site, we mapped insert sequences beginning and ending with the first and last base pair of the BsaI binding sites and allowed up to three mismatches and only kept reads that were the expected 47 bp. We counted the frequency of errors in the BsaI and GG site.

### PacBio sequencing for UniProt GFP and SynGFP GG assemblies

We applied long-read PacBio sequencing to the SynGFP, SynGFP B, and UniProt GFP assemblies to assess assembly fidelity. For each library, the assembly product was transformed into NEBExpress I^q^ competent cells (New England Biolabs, no. 3037I), and the full transformation, minus a small volume to measure transformation efficiency, was plated onto Luria broth (LB) + carbenicillin (carb) plates. Plates were incubated overnight at 37°C and then moved to 4°C for another 24 hours. After this, an equal fraction of cells from each plate was collected and added to 50 ml of growth media (25 g/liter LB, 10 g/liter molecular-grade agar, and 1% glucose) and grown until ~1 optical density at 600 nm (OD600). We harvested the DNA using a plasmid prep kit (QIAGEN, no. 12943), linearized the DNA using Not I–HF (New England Biolabs, no. R3189) following the manufacturer’s protocol, and submitted samples to the University of Wisconsin—Madison Biotechnology Center. Sequencing achieved at least 16× coverage based on the number of unique transformants.

We used “pbmm2” to map library sequences to full-length PacBio reads using HiFi as presets. Library sequences included ~25-bp upstream and downstream flanks of the destination vector sequence to ensure that assembled sequences and plasmid were contiguous. We kept reads that satisfied the following criteria: (i) Library sequence was contiguous with the destination vector, (ii) the full-length plasmid read was within 100 bp of the predicted read length to ensure that the plasmid did not have multiple assembled sequences, (iii) the aligned protein sequence was within 98% of the predicted library sequence to filter out sequences with inserted or missing fragments, and (iv) the mean quality score for aligned bases is ≥30 with an overall map quality of 60. We categorized mapped reads as correct if the sequence perfectly matched the designed sequence and considered sequences observed if a perfect sequence match was present at least once. Sequences with up to five mismatches and zero insertions and deletions were used for additional synthesis error analysis to calculate the fraction of sequences that were assembled without small errors introduced through synthesis, PCR, or sequencing.

We further identified a subset of incorrect assemblies in SynGFP that we use as nonfluorescent controls in our characterization of the SynGFP library. We looked for sequences between the vector ligation sites that occurred more than 100 times that did not match any known library sequences. We selected a subset of 113 of these to create a set of negative sequences as nonfluorescent controls.

### Experimental evaluation of SynGFP sequences

We measured GFP fluorescence for the SynGFP library using FACS as previously described by Sarkisyan *et al.* (*5*). Briefly, the SynGFP library was transformed into NEBExpress I^q^ competent cells (New England Biolabs, no. 3037I) following the manufacturer’s protocol and plated on LB-carb agar plates [25 g/liter LB (Dot Scientific Inc., no. D5L24400-2000) and 10 g/liter molecular-grade agar]. Plates were incubated overnight at 37°C and then incubated for 24 hours at 4°C to allow for expression and fluorescence maturation. Plates were washed with 4 ml of LB + carb (25 g/liter LB and 100 μM carbenicillin) and used to create 1 OD expression cultures. Duplicate cultures were induced with 1 mM isopropyl β-d-1-thiogalactopyranoside and incubated for 2 hours (23°C, 230 rpm). We spiked in wild-type avGFP such that it was 1% of the total library. After incubation, 1 OD of cells were spun down and resuspended in ice-cold phosphate buffer (1× phosphate-buffered saline).

We sorted GFP variants based on fluorescence intensity. We used ex561/em670 and ex488/em530 wavelengths to detect mKate2 and GFP fluorescence, respectively. Compensation was applied to remove overlapping signal. To account for variations in expression, we first defined a narrow gate for mKate2 expression and then defined a series of seven sort gates for GFP expression within this population. The GFP gates followed an approximately uniform distribution across the logarithmic scale with slightly larger gates for the least and most fluorescent populations. Sorted cell populations were grown to 0.6 to 1.0 OD in LB-carb media with 1% glucose to suppress protein expression and reduce growth bias due to protein expression. The DNA was harvested from 50-ml cultures using a QIAGEN Plasmid Plus Midi Kit (QIAGEN, no. 12943).

Sorted FACS populations were sequenced using Oxford Nanopore and used to calculate a LogF value for all SynGFP sequences and the incorrect assemblies. We used “pbmm2” to map reads to library sequences. The DNA was linearized using tagmentation, which may cleave a single plasmid at multiple points. We could not use read length to filter reads as with our PacBio samples and instead only required that mapped library sequences were contiguous with the destination vector, overlapped by at least 25 bp, and were not longer than the maximum predicted read length. We additionally applied the following filters: (i) The aligned protein sequence length was within 98% of the predicted length, (ii) the mean quality score of aligned bases was ≥30, and (iii) the map quality was 60. Library sequences were very diverse with the closest related sequences sharing ~90% sequence identity. Assembly fragments also varied in size, which would create significant size disparity in the event of incorrect assemblies. We reasoned that incorrect sequences would vary significantly from designed sequences and allowed mapped reads to have up to five mismatches and six insertions and deletions.

Last, we filtered sequences for synthesis errors by removing ONT sequences with known or novel point mutations at positions where we observed synthesis errors in the PacBio data. For each sequence, we aligned mapped ONT reads with the mapped PacBio reads described previously. Synthesis errors were identified as positions where at least one read reported a point mutation with a qscore ≥80. We removed any reads with incorrect bases at these positions, but otherwise permitted some errors in other portions of the sequence. The PacBio sequencing has greater library coverage than the Oxford Nanopore sequencing, and we reasoned that Oxford Nanopore sequencing would not observe any novel point mutations.

We calculated a LogF value for each sequence based on its probability distribution across GFP bins following previously described methods (*43*). We excluded sequences from analyses if they had fewer than 55 combined counts across bins in either replicate. We report LogF values as an average of replicate LogF scores since there was a strong correlation between replicates (Pearson’s ρ: 0.94).

## Supplementary Materials

### The PDF file includes:

Supplementary Information Figs. S1 to S17 Tables S1 to S3 Legend for data S1 References
