# Supplementary material for: Scalable and cost-efficient custom gene library assembly from oligopools
Source: Sci Adv. 2026 May 22;12(21):eady2279. doi: 10.1126/sciadv.ady2279 (PMC13196743; doi:10.1126/sciadv.ady2279)
Supplement: Supplementary file 1 — Supplementary Information Figs. S1 to S17 Tables S1 to S3 Legend for data S1 References [file sciadv.ady2279_sm.pdf]

Science Advances
AAAS

Supplementary Materials for

Scalable and cost-efficient custom gene library assembly from oligopools

Chase R. Freschlin *et al.*

Corresponding author: Philip A. Romero, philip.romero@duke.edu

Sci. Adv. **12**, eady2279 (2026)
DOI: 10.1126/sciadv.ady2279

The PDF file includes:

Supplementary Information
Figs. S1 to S17
Tables S1 to S3
Legend for data S1
References

Other Supplementary Material for this manuscript includes the following:

Data S1

SUPPLEMENTARY INFORMATION

OMEGA design method pseudocode

Below is a step-by-step overview of the OMEGA oligonucleotide design process. This pseudocode outlines the algorithmic structure used to design fragmented gene assemblies from input sequences. A full implementation is available at github.com/RomeroLab/omega.

1. **Input target genes and parameters.** The user provides a list of target DNA sequences, the desired number of Golden Gate (GG) sites per subpool (`njunctions`), upstream/downstream fixed GG overhangs for vector cloning, and optimization run parameters including the number of optimization steps (`nopt_steps`) and the number of independent optimization runs (`nopt_runs`).

2. **Estimate required fragments per gene.** OMEGA computes how many 300 bp oligos (fragments) are needed to cover the length of the longest gene. This accounts for primer binding sites and cloning sites, leaving 246 bp to be used for gene sequences. All genes are broken into the same number of fragments using this number and determines how many GG sites are needed per gene.

3. **Determine the number of subpools.** Determine how many subpools are required considering the total number GG sites needed to assemble all target genes and `njunctions`. We provide suggested primer pairs for up to 92 subpools.

4. **Group genes into subpools.** The genes are randomly and uniformly distributed amongst subpools such no subpool uses more than the specified number of GG sites (`njunctions`) including vector ligation sites.

5. **Optimize each subpool independently.** Subpool optimization designs the GG sites used to fragment each gene in the subpool. For each subpool:

    a. **Design GG sites.** OMEGA performs several independent design runs specified by `nopt_runs` using simulated annealing. Each run has the chance to find a different set of high-fidelity GG sites. For each design run:

        i. **Identify valid fragmentation sites.** Each position in the gene corresponds to the start index of a unique GG site since both the GG site sequence and position are important for gene fragmentation. For each gene, a sliding window of 4 bp is used to identify the index and sequence of all possible GG sites within the gene.

        ii. **Initialize optimization run with uniformly spaced sites.** Each gene is broken into evenly spaced fragments to initialize a random set of GG sites. If all sites are not unique, break points are iteratively shifted +/- 1 bp until all unique sites are achieved.

        iii. **Predict assembly fidelity of initial set of GG sites.** The fidelity of a subpool is calculated as in Pryor et al. (*19*), shown here for clarity. $F = p(O_1) \times p(O_2) \times p(O_n)$ where $p(O_i)$ is the number of correct ligations divided by the number of incorrect ligations for a GG site and its Watson Crick pair.

        iv. **Run GG site optimization for `nopt_steps`.** A decreasing logarithmic temperature gradient is used to guide the optimization of GG sites. For each step in the temperature gradient:

            1. **Randomly change GG site.** From the current set of sites, choose one site randomly and exchange it for another valid site that 1) is not used in the subpool and 2) maintains oligo length constraints.

2. **Predict assembly fidelity.** Predict the fidelity of new GG set of GG sites following fidelity calculation in step 5.a.iii.
3. **Accept or reject GG set.** If the new set has a higher predicted fidelity, the new set is accepted. If the set is worse, then accept or reject depending on the temperature gradient $X > e^{\Delta F/T}$ where X is randomly generated such that $0.0 \leq X < 1$.

v. **Save highest fidelity set of GG sites observed during optimization run.** This represents one optimal solution for subpool design.

b. **Select highest fidelity set achieved over all optimization runs for each subpool.**

6. **Construct fragment sequences.** For each subpool, sequences are broken at designed GG site junctions. Cloning sites and primer binding sites are added and optional padding basepair padding is added between the cloning and primer site to make all oligos the specified length.

7. **Output files.** The following data are output for the user.

a. Optimization results. This file contains the original sequences, fragmented oligo sequences, fidelity predictions, GG site information, subpool information for all sequences in the library, and other relevant information.

b. Pool statistics. This file provides a high level field of the pools in the library, the number of genes they contain and GG sites they use, predicted fidelity, primer binding sites and name, and other relevant information.

c. Oligo sequences. This file contains the designed sequences ready to order from a commercial supplier such as Twist Biosciences.

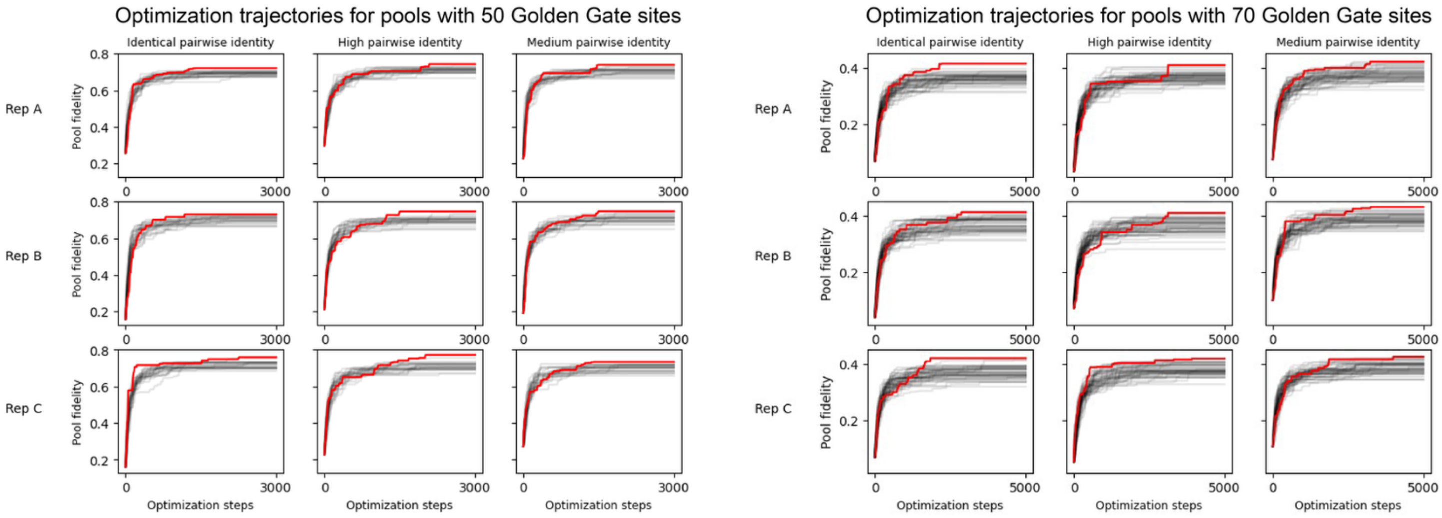

Supplementary Figure 1. Robustness and convergence of the simulated annealing (SA) optimization in OMEGA. Each panel shows the predicted fidelity score across 25 independent SA runs for one of nine gene libraries spanning high, medium, and identical sequence identity levels, optimized using either 50 or 70 GG sites per pool. The plots demonstrate substantial increases in predicted fidelity across iterations and strong convergence across independent runs, supporting the robustness of the optimization strategy.

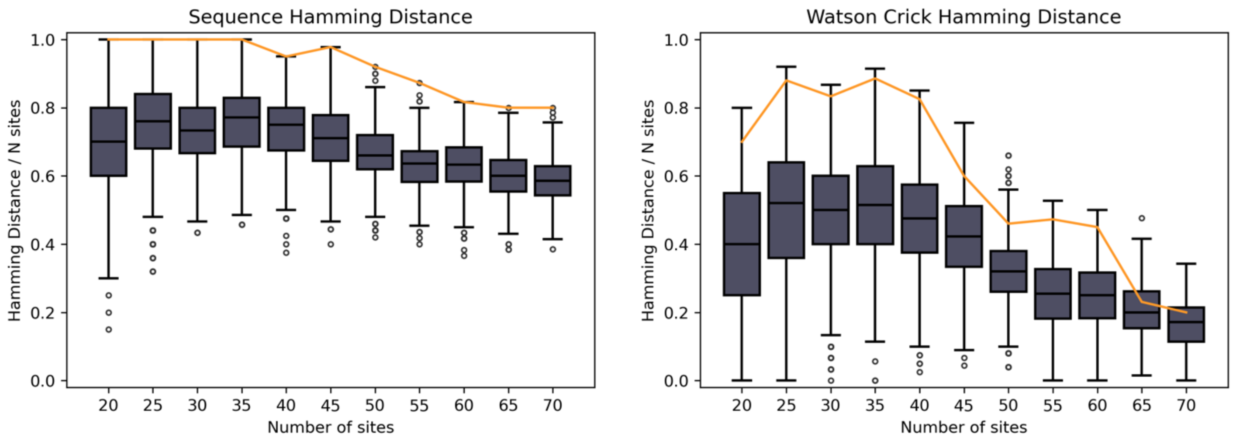

Supplementary Figure 2. Pairwise hamming distance of optimized Golden Gate sets using 20 to 70 sites. Boxplot shows pairwise HDs for all designed sets, orange line shows the pairwise HD for the two experimentally validated sets. **(a)** shows diversity in terms of sequence identity and **(b)** shows diversity in terms of Watson Crick pair.

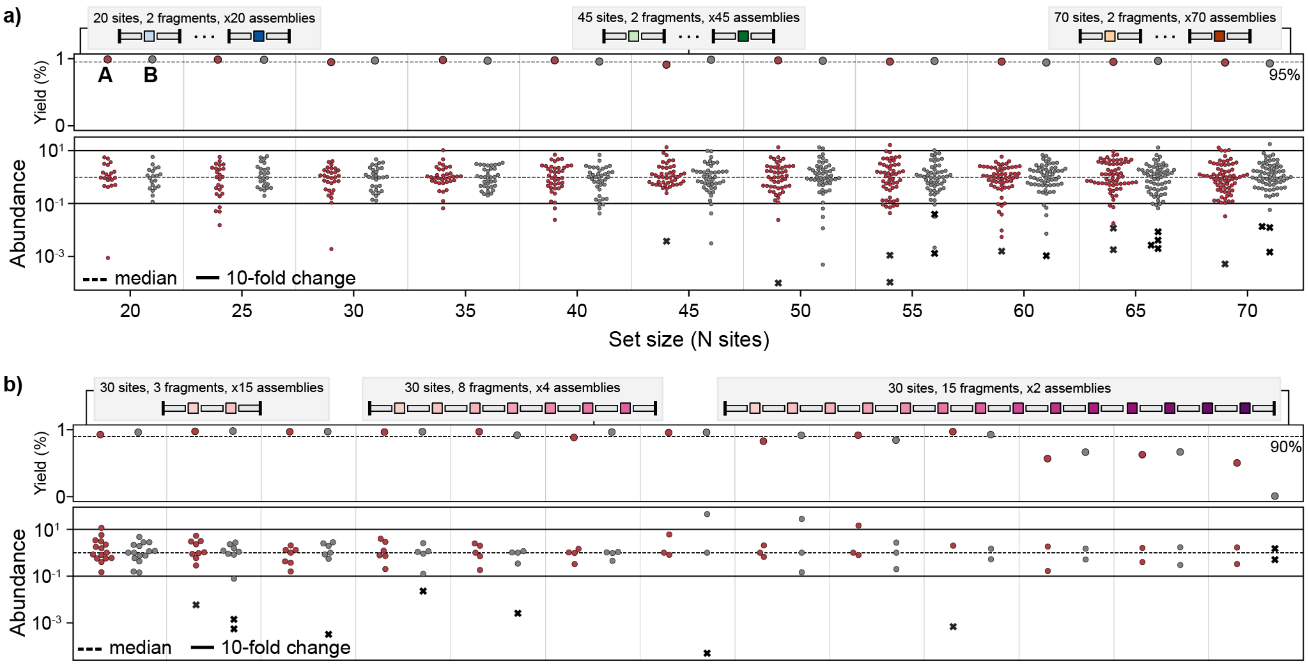

Supplementary Figure 3. Fidelity of GG assemblies with different numbers of GG sites and fragments per assembly including constructs with BsaI. Replicate assemblies were performed for each GG set size or fragment number. Replicates are marked in red or grey. Constructs containing a BsaI site in the assembled sequence are marked with a black X. All statistics include reads where constructs contain BsaI in the assembled sequence. Constructs containing BsaI are under-represented in the GG assembly. **(a)** Assemblies using 20 to 70 GG sites. **(b)** Assemblies using 3 to 15 fragments.

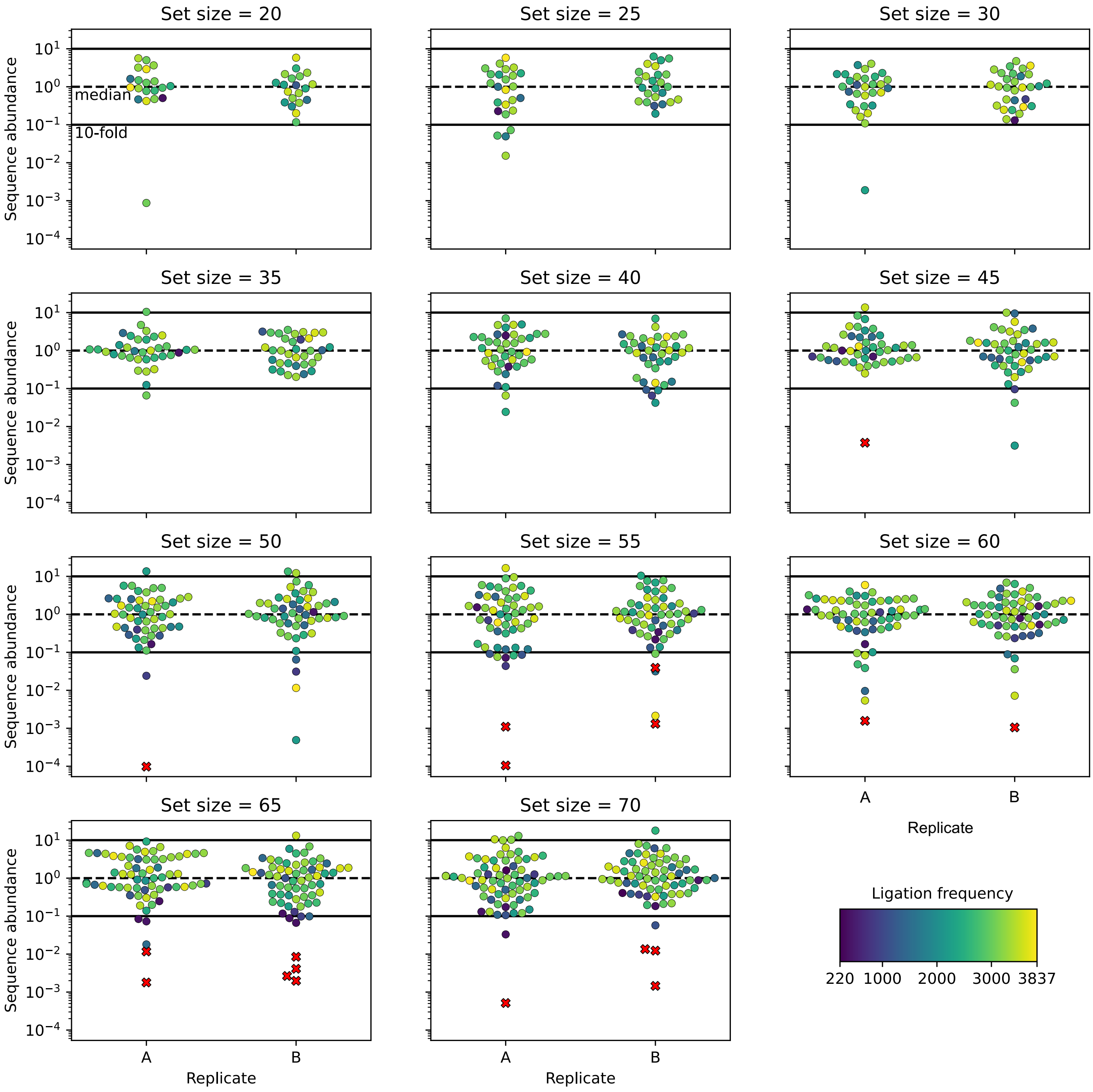

Supplementary Figure 4. Minimum ligation frequency for assemblies using 20 to 70 Golden gate sites plotted by abundance. Potapov et al. (*20*) estimate ligation efficiencies of individual Golden Gate sites as the number of correct ligation events in their ligation frequency experiments. High and low ligation frequencies correspond to high and low ligation efficiencies. Here, assembled sequences are plotted for all replicates for each set size according to the x-fold sequence abundance. The hue corresponds to ligation frequency for of the designed GG site connecting the two fragments. Low and high-abundant sequences contain low-efficiency sites. Low-efficiency sites may be more common in low-abundant sequences for assemblies using more GG sites. Red Xs mark constructs with BsaI sites in the assembled sequence.

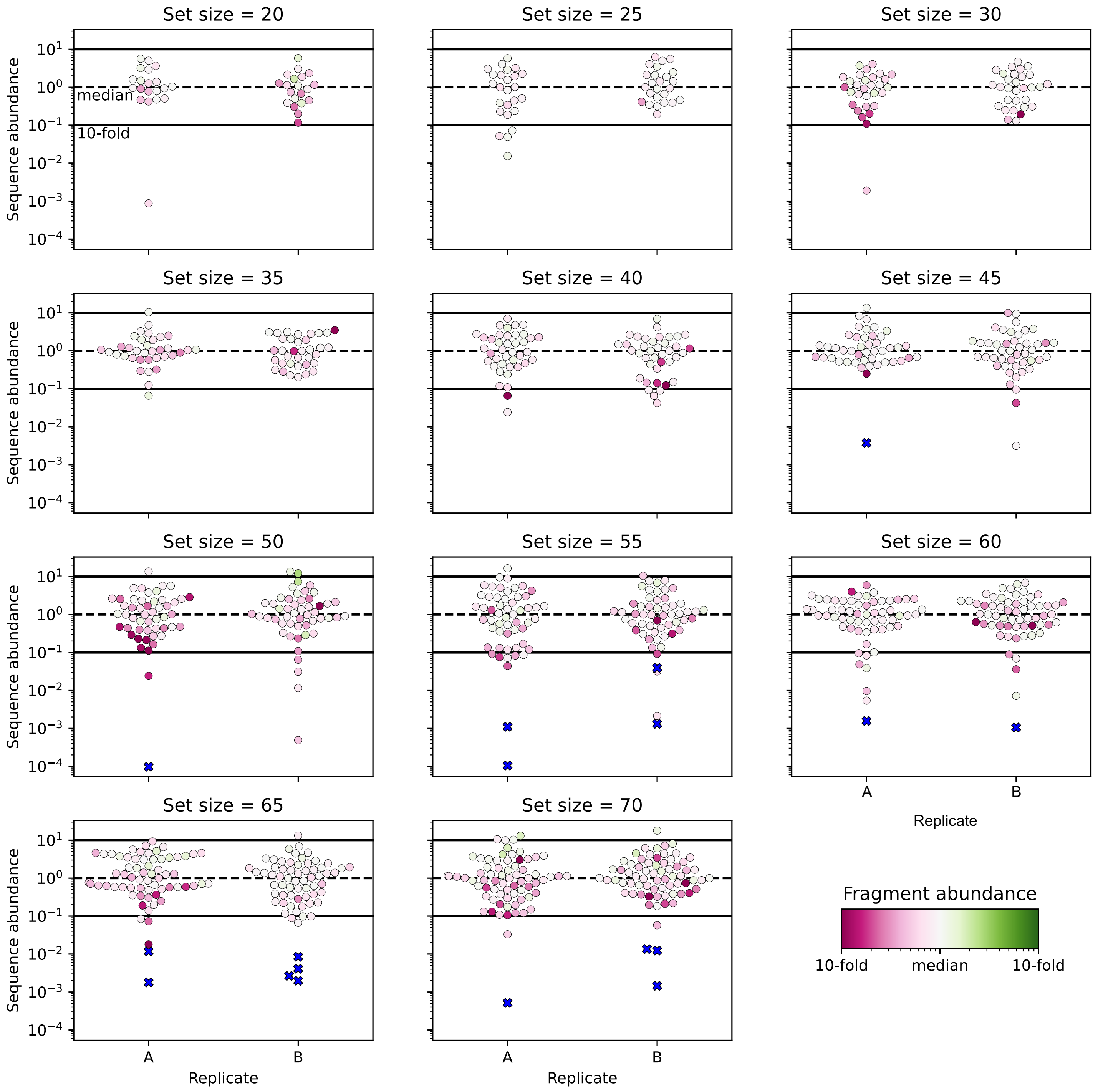

Supplementary Figure 5. Oligo abundance for assemblies using 20 to 70 Golden gate sites plotted by abundance. We sequenced the amplified oligos used as input to the GG assembly and calculated the relative abundance of each fragment with respect to the median abundant fragment for each assembly pool. Here, assembled sequences are plotted for all replicates for each set size according to the x-fold sequence abundance. The hue corresponds to the least abundant fragment used in the assembly. In larger assemblies using more than 45 sites, assemblies with low-abundant fragments tend to result in low-abundant assemblies. Blue Xs mark constructs with BsaI sites in the assembled sequence.

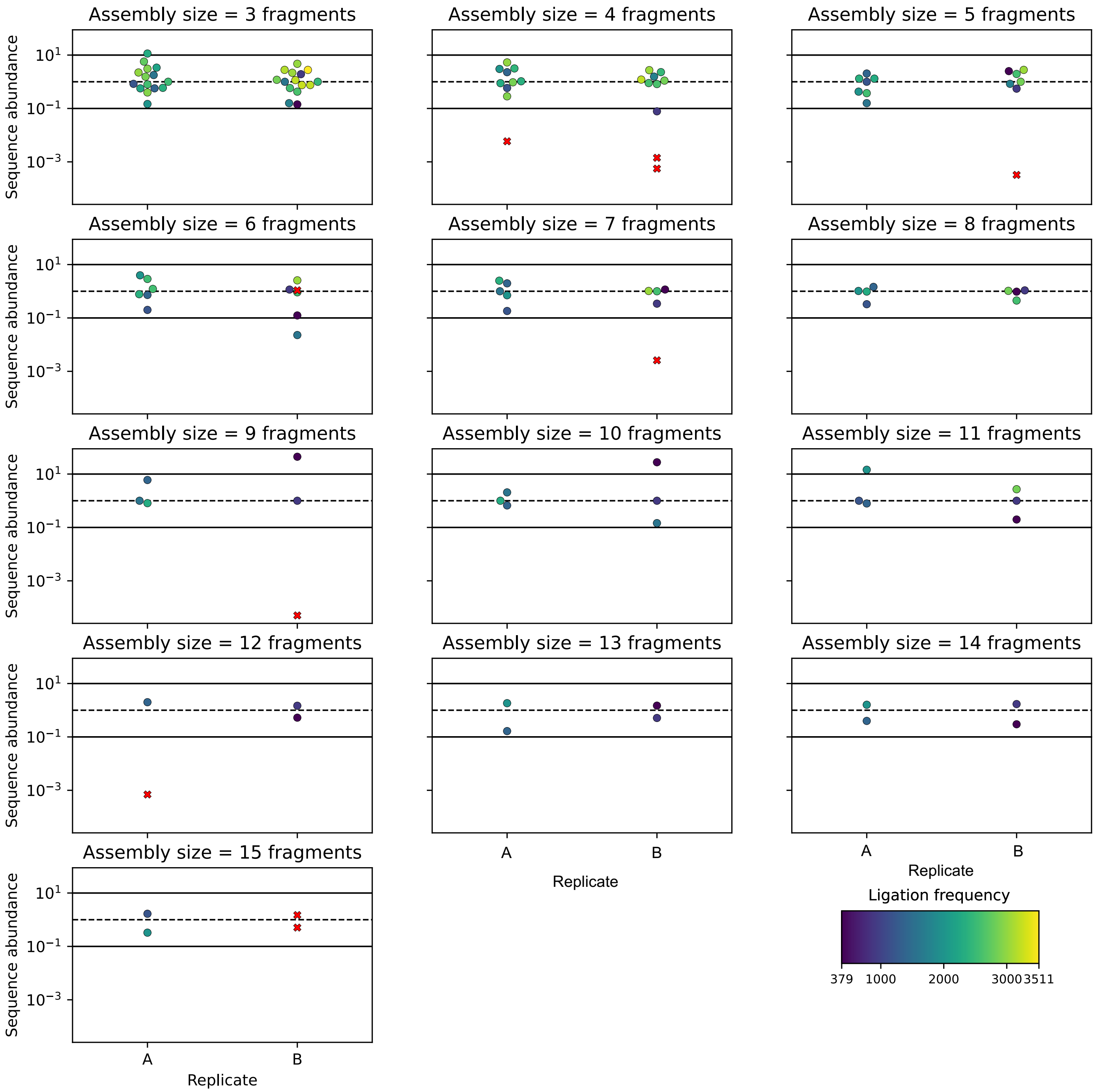

Supplementary Figure 6. Minimum ligation frequency for assemblies using 3 to 15 fragments plotted by abundance. Potapov et al. (*20*) estimate ligation efficiencies of individual Golden Gate sites as the number of correct ligation events in their ligation frequency experiments. High and low ligation frequencies correspond to high and low ligation efficiencies. Here, assembled sequences are plotted for all replicates for fragment number according to the x-fold sequence abundance. The hue corresponds to ligation frequency for of the lowest efficiency GG site. Red Xs mark constructs with BsaI sites in the assembled sequence.

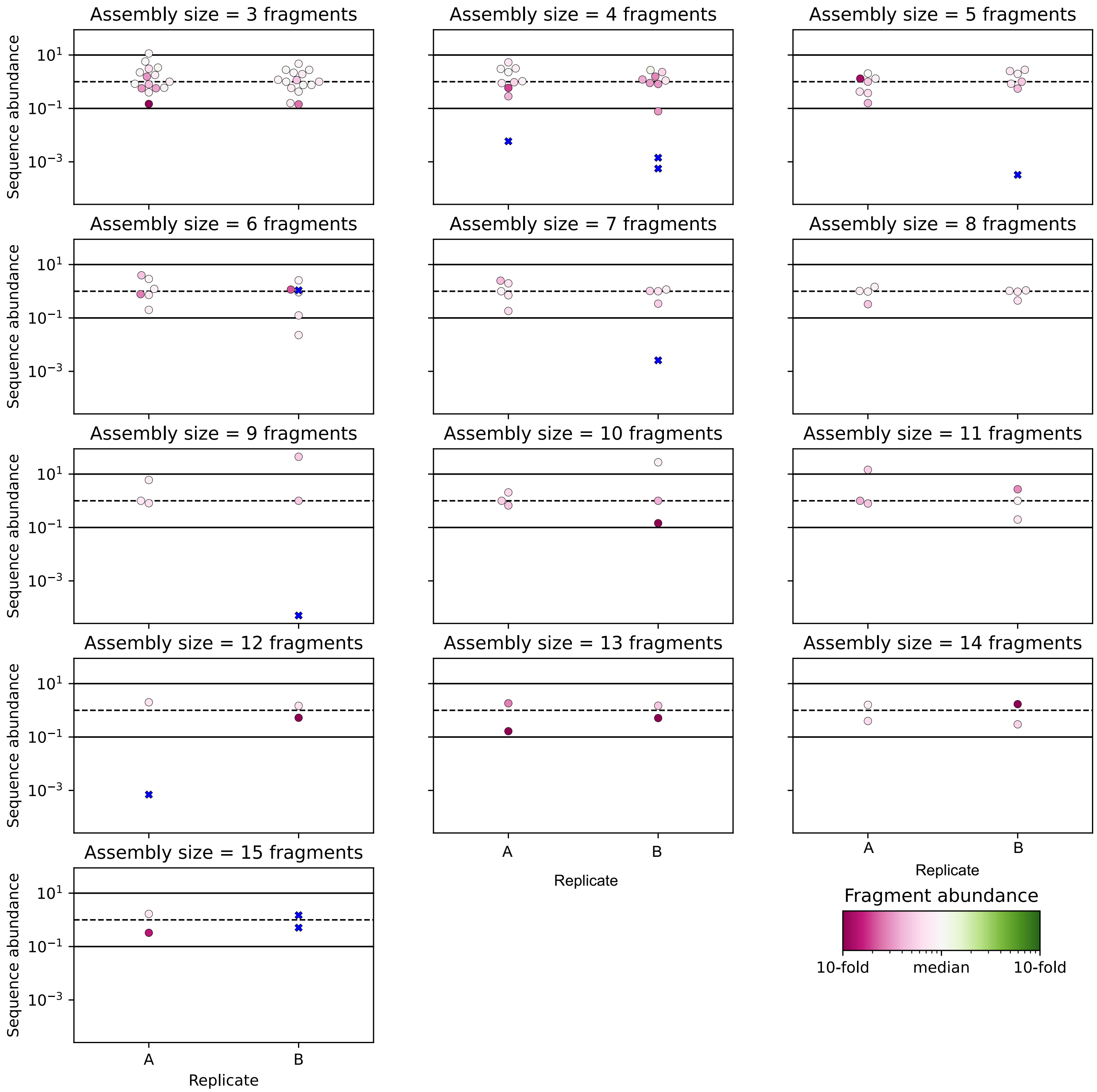

Supplementary Figure 7. Oligo abundance for assemblies using 3 to 15 fragments plotted by abundance. We sequenced the amplified oligos used as input to the GG assembly and calculated the relative abundance of each fragment with respect to the median abundant fragment for each assembly pool. Here, assembled sequences are plotted for all replicates for each fragment number according to the x-fold sequence abundance. The hue corresponds to the least abundant fragment used in the assembly. Low-abundant fragments appear to be slightly more common in low-abundant sequences. Blue Xs mark constructs with BsaI sites in the assembled sequence.

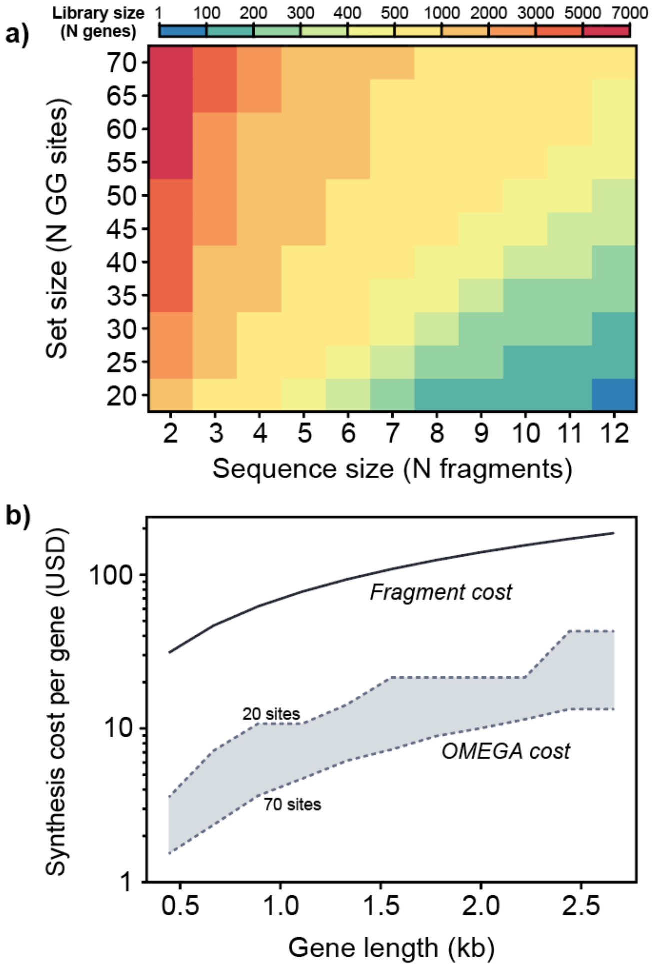

Supplementary Figure 8. OMEGA assembles hundreds of genes and achieves > 10-fold cost reduction compared to fragment synthesis. (a) Library size depends on the number of GG sites, sequence length, and the number of subpools. The set of orthogonal primers developed by Subramanian et al. (*22*) can index up to 92 subpools using unique pairs or 185 using a unique reverse primer and universal forward primer. We use 96 subpools to estimate library size to represent the throughput of a standard 96-well plate. **(b)** Individual costs for sequences (0.3-2.6 kb) synthesized as fragments or assembled from an oligopool using Twist Bioscience pricing. Oligopool gene costs are calculated by dividing the total pool costs by the number of genes in the OMEGA library. We show the range of costs for library sizes using 96 subpools and between 20 and 70 sites per assembly. These cost estimates are intended as rough benchmarks to contextualize OMEGA's potential savings. They assume that all target constructs are successfully recovered from the pooled assembly. In practice, incomplete recovery of some designs reduces the realized cost benefit. Additionally, the fragment synthesis prices reflect clonal constructs; isolating individual clones from an OMEGA library increases the per-gene cost.

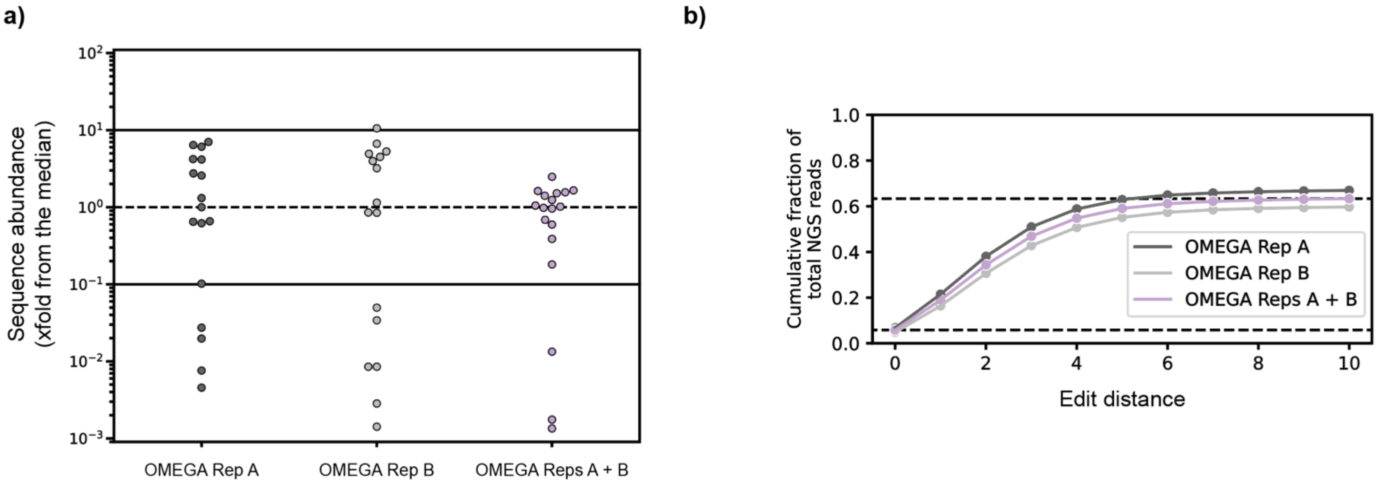

Supplementary Figure 9. Sequence abundance of individual and combined Cas9 libraries. We used OMEGA to find two GG solutions for 18 Cas9 sequences distributed across 3 subpools (Rep1, Rep2). **(a)** Sequence abundance after OMEGA assembly. We assembled, pooled, and sequenced the two libraries independently and calculated the abundance of sequence-perfect assemblies (perfect codon match) as fold change from the median abundant sequence. The counts of sequence-perfect constructs were normalized to 100k and added together to estimate the sequence abundance if assemblies were pooled between libraries. The combined library has improved coverage and more sequences that fall within tenfold of the median abundant sequence than the individual libraries. **(b)** Edit distance of assemblies in individual and combined libraries. Edit distance is the number of mismatches, insertions, and deletions necessary to make two sequences equal to one another. To combine counts, the counts for individual libraries were first normalized to 100k based on the total number of NGS reads and then combined by edit distance. Edit distance is plotted as the cumulative fraction of all NGS reads.

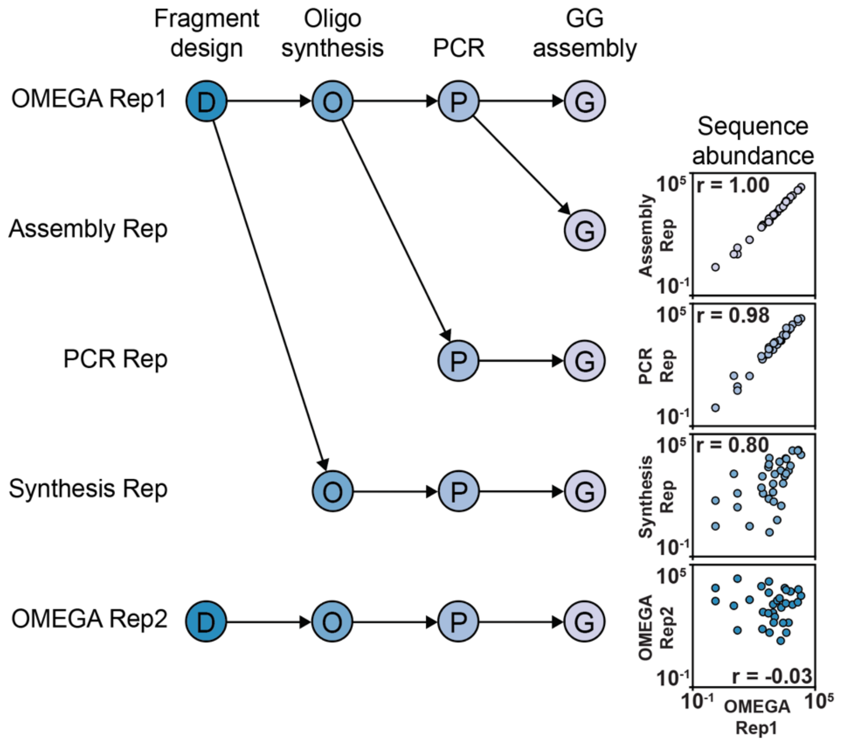

Supplementary Figure 10. Replicate assemblies of thirty-three rubisco sequences. OMEGA Rep1 represents a full design run with all computational design steps and experimental assembly steps. Each replicate branches from OMEGA Rep1 design at different steps to investigate sources of bias. Assembly Rep repeats the OMEGA Rep1 GG assembly, PCR Rep repeats the subpool and PCR and GG assembly, Synthesis Rep repeats oligo synthesis, PCR, and GG assembly, and OMEGA Rep2 is a replicate of the full design workflow. Correlation between sequence abundance is weaker for replicates that branch earlier in the design workflow.

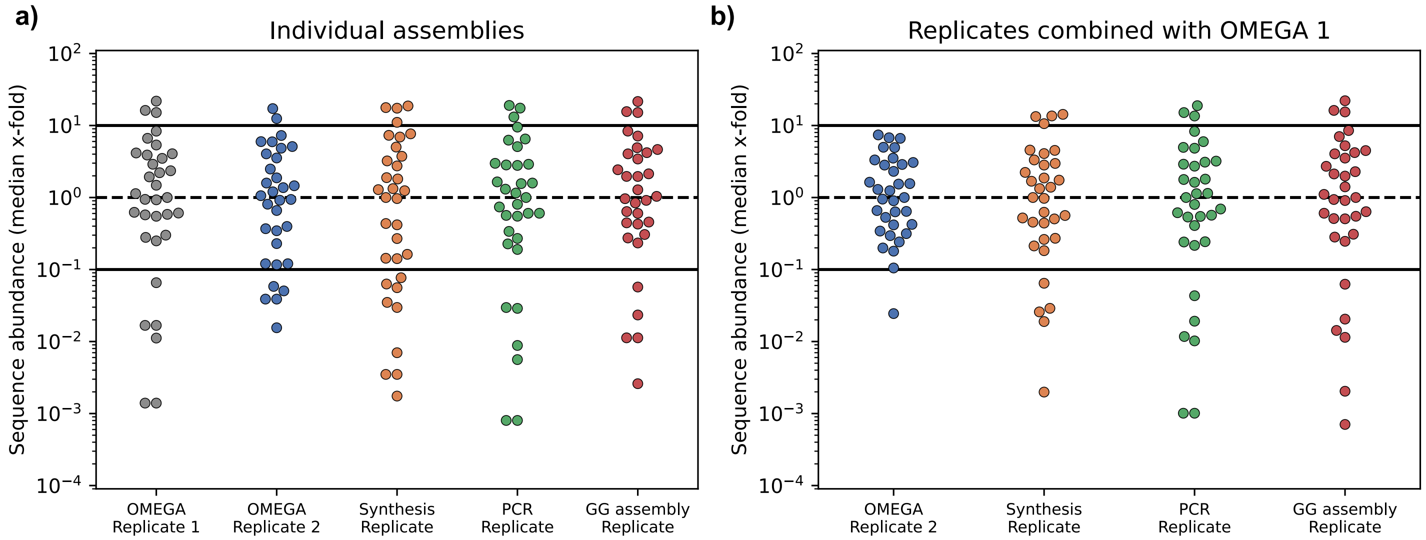

Supplementary Figure 11. Sequence abundances of replicate rubisco assemblies. Each replicate assembles the same 33 sequences level distributed across 3 subpools. All abundances are represented as foldchange from the median abundant sequence. The dashed line represents the median and solid lines represent tenfold above or below the median. **(a)** Sequence abundance for individual replicate assemblies. OMEGA Replicate 1 and 2 use different GG sites, the synthesis replicate uses independently synthesized oligos identical to OMEGA Replicate 1, the PCR replicate uses OMEGA Replicate 1 oligos from a duplicate subpool PCR, and the GG assembly replicate uses the same subpool PCR used in OMEGA Replicate 1 to perform a duplicate GG assembly. **(b)** We simulated combining products across replicates by normalizing counts for individual assemblies to 100k and adding counts together by sequence identity. The foldchange of sequence abundance was calculated from the combined counts. The sequence abundance of combined OMEGA replicates has fewer under- and over-represented sequences than either of the individual libraries. The combined libraries of all other replicates have the same number of under- and over-represented sequences as at least one of the individual libraries.

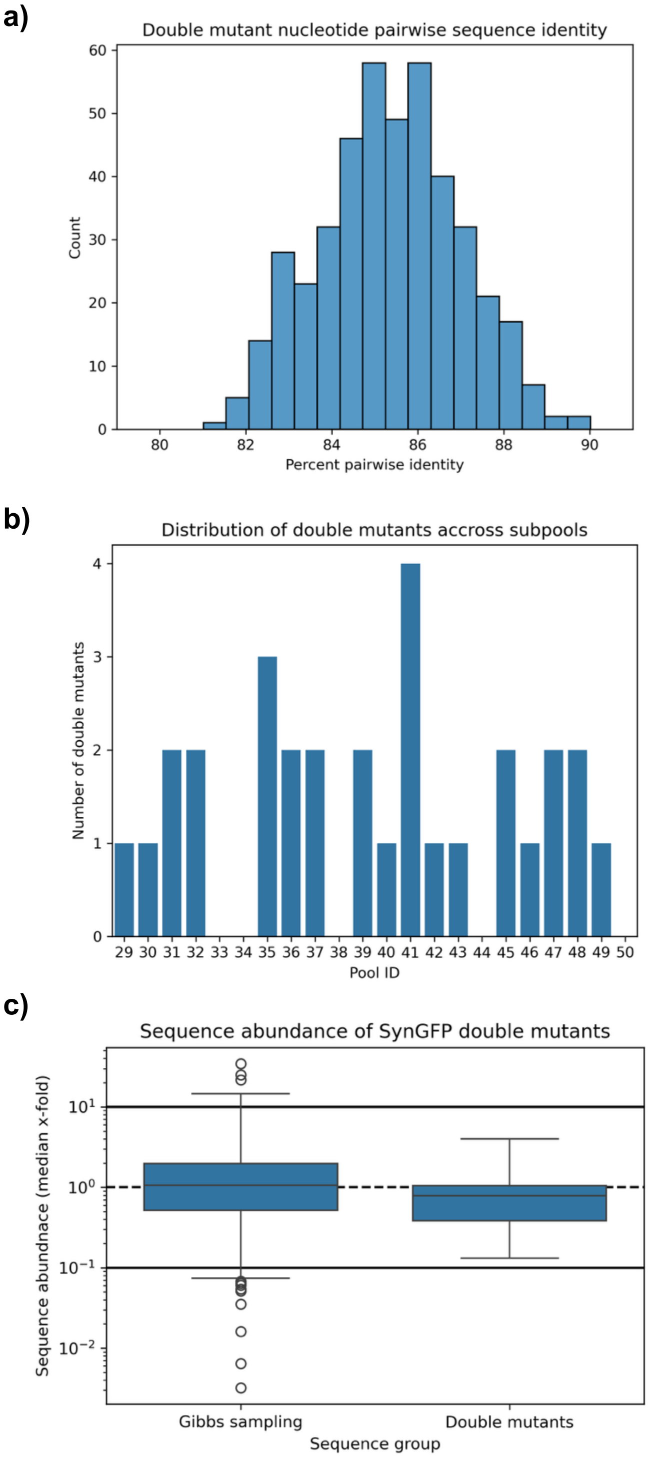

Supplementary Figure 12. Double mutant sequence identity and abundance in SynGFP library. (a) Pairwise nucleotide sequence identity of double mutants. Pairwise identity is the fraction of shared nucleotides relative to sequence length. **(b)** Distribution of double mutants in SynGFP pools. Most commonly pools have one or two double mutants with some having none. **(c)** Sequence abundance of SynGFP variants designed using Gibbs sampling (N=307) and double mutants (N=29).

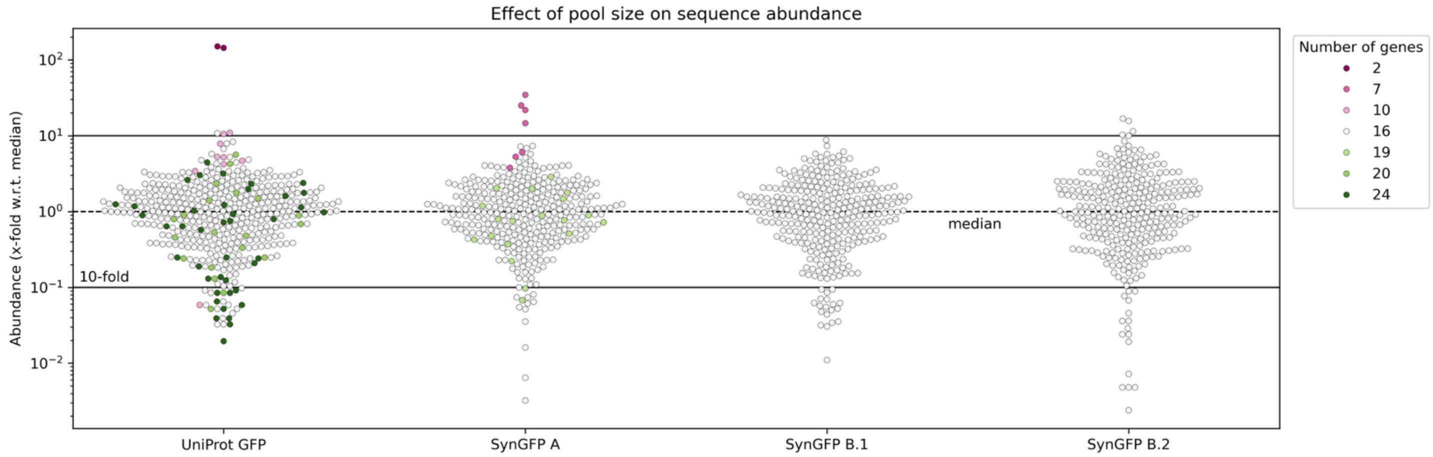

Supplementary Figure 13. Varying the number of sequences in sub-assembly pools biases library sequence distribution. Library assemblies were sequenced with long read PacBio sequencing and plotted as the x-fold with respect to the median-abundant sequence for each library. Color indicates the size of subassembly pools (ex. white indicates that sequence was assembled with 15 other sequences for a total pool size of 16). Sequences from small assembly pools (<16 sequences) are more abundant than larger assemblies. Large assemblies (>=20 sequences) may result in under-represented sequences.

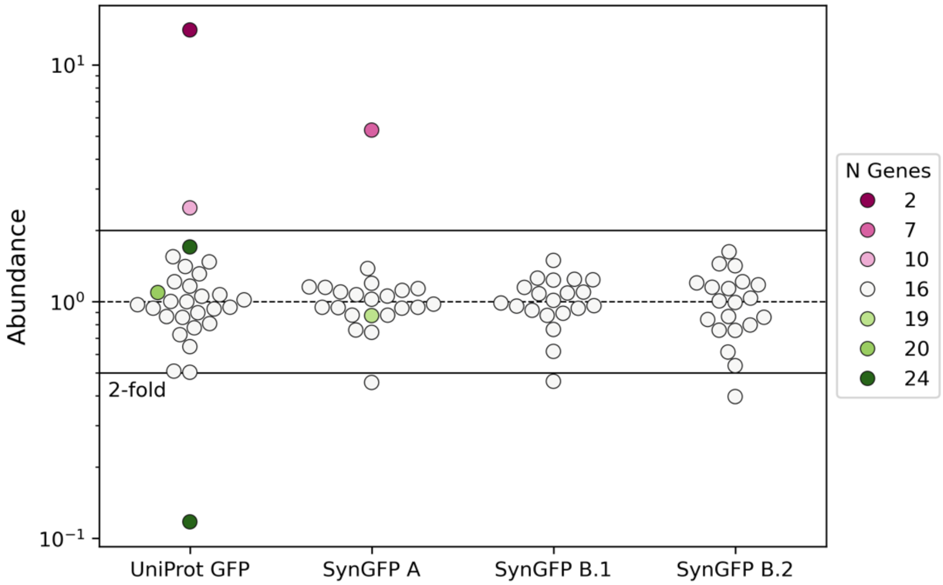

Supplementary Figure 14. Assembly uniformity across subassembly pools. We assessed if all sub-assembly pools were equally represented in the combined library. We combined assembly products from all pools for each library without normalization and applied long read PacBio sequencing. The total number of reads were counted for each pool and divided by the median-abundant sub-assembly pool to represent pool abundance as x-fold from the median. Sub-assembly pools are colored by the number of genes assembled in the pool. Most pools assemble within 2-fold of the median. Pools that assemble fewer genes are typically over-abundant in the library (UniProt GFP and SynGFP A). Removing pools with fewer genes mitigates assembly bias (SynGFP A vs. SynGFP B).

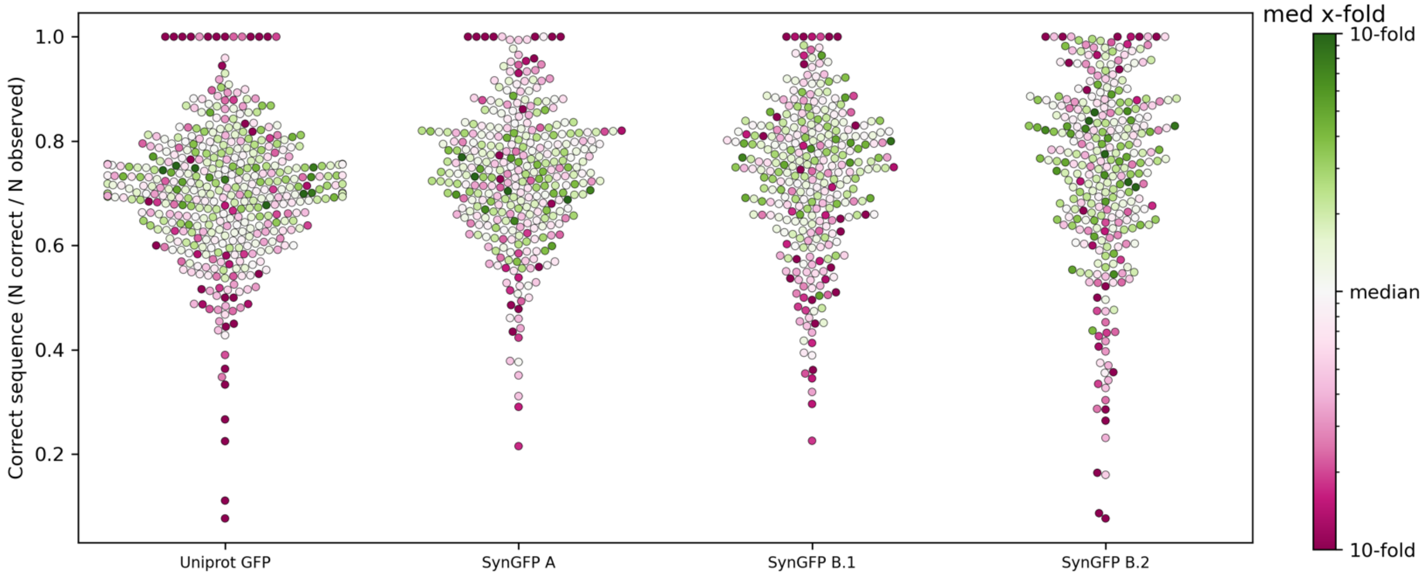

Supplementary Figure 15. Fraction of correct assemblies without synthesis error. Due to oligo synthesis error, correct assemblies with all fragments assembled in the correct order may contain sequence errors. To assess the fraction of assemblies that are also sequence-correct, we used pbmm2 to align designed library sequences against PacBio reads. We counted all reads that aligned with <= 5 mismatches to a library sequence and excluded any reads with insertions or deletions. The subset of these reads that perfectly match the designed sequence define the fraction of correct sequences. Sequences are plotted by the fraction of correct reads and colored by its abundance (x-fold from the median-abundant sequence) on a log scale. More than 60% of mapped reads are error-free for abundant sequences (>= median abundant sequence).

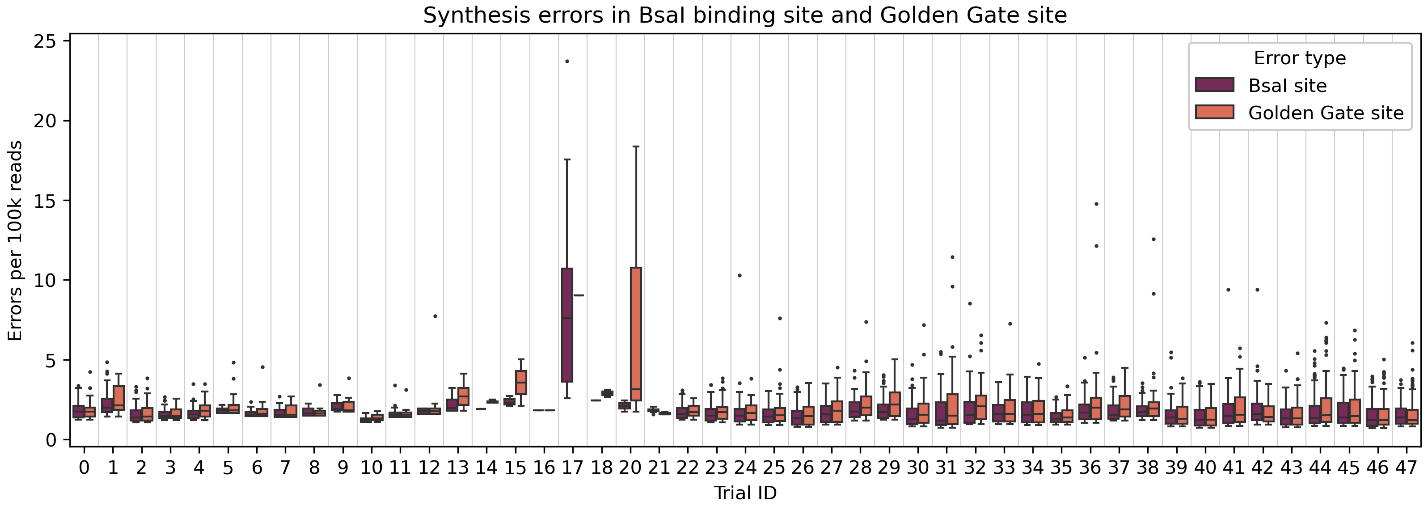

Supplementary Figure 16. Synthesis errors in the BsaI binding site or Golden Gate site. We applied Illumina sequencing for amplified oligos from parameterization assemblies. We report errors as the total number of reads normalized to 100k that contain synthesis errors in either the BsaI binding site or Golden Gate site. In most cases, synthesis errors in the BsaI and GG sites occur in < 0.005% of reads.

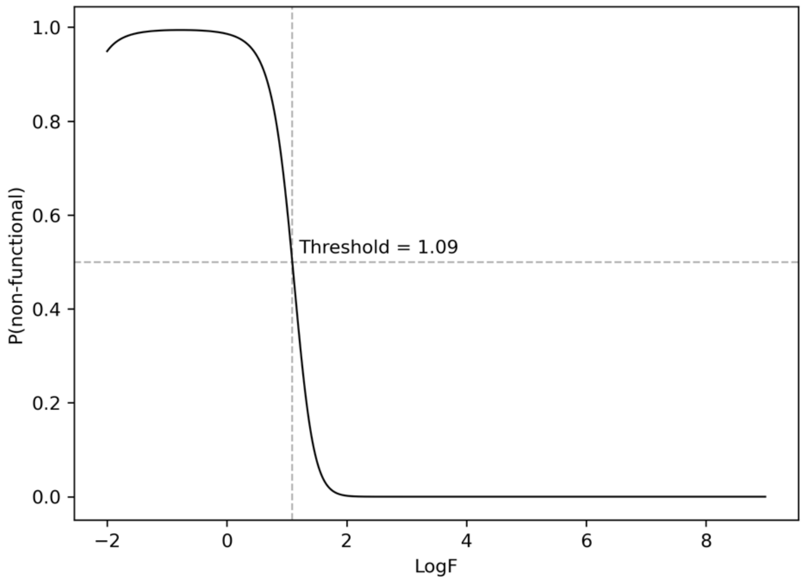

Supplementary Figure 17. LogF functional cutoff for GFP fluorescence. A Gaussian Mixture Model from the scikit-learn package (44) was fit to designed sequences with >= 55 cumulative counts across sorted populations (N=314). We used LogF values averaged across two experimental replicates. We plotted P(non-functional) for LogF values in increments of 0.01 from -2 to 9. The LogF closest to 0.5 was used as the functional cutoff.

Table S1. Number of sequences used in experiments varying number of Golden Gate sites. Set size corresponds to the number of GG sites used in a single assembly pool. For each set size and replicate, the number of total designed sequences is reported and the number of sequences without BsaI in the assembled sequence is reported in parentheses.

Number of GG sites	Replicate	Designed sequences (without BsaI)
20	A	20 (20)
	B	20 (20)
25	A	25 (25)
	B	25 (25)
30	A	30 (30)
	B	30 (30)
35	A	35 (35)
	B	35 (35)
40	A	40 (40)
	B	40 (40)
45	A	45 (44)
	B	45 (45)
50	A	50 (49)
	B	50 (50)
55	A	55 (53)
	B	55 (53)
60	A	60 (59)
	B	60 (59)
65	A	65 (63)
	B	65 (61)
70	A	70 (69)
	B	70 (66)

Table S2. Number of sequences used in experiments varying fragment number. Assembly size corresponds to the number of fragments needed to encode each designed sequence. For each assembly size and replicate, the number of total designed sequences is reported and the number of sequences without BsaI in the assembled sequence is reported in parentheses.

Assembly size	Replicate	Designed sequences (without BsaI)
3	A	15 (15)
	B	15 (15)
4	A	10 (9)
	B	10 (8)
5	A	7 (7)
	B	7 (6)
6	A	6 (6)
	B	6 (5)
7	A	5 (5)
	B	5 (4)
8	A	4 (4)
	B	4 (4)
9	A	3 (3)
	B	3 (2)
10	A	3 (3)
	B	3 (3)
11	A	3 (3)
	B	3 (3)
12	A	2 (1)
	B	2 (2)
13	A	2 (2)
	B	2 (2)
14	A	2 (2)
	B	2 (2)
15	A	2 (2)
	B	2 (0)

Table S3. Design statistics reporting number of observed and functional sequences in SynGFP. Designed indicates the number of sequences included in the library. "> 55 counts" is the number of sequences observed >= 55 times across all sort bins. N functional are the number of sequences above the LogF cutoff 1.09. Fraction functional is the fraction of observed designs that are functional.

Population	Designed	> 55 counts	N Functional	Fraction functional
Negative	-	76	2	.03
MIFST-doubles	15	15	12	.80
CARP-640M-doubles	15	14	5	.36
MIFST-MCMC	100	85	0	0
CARP-MCMC	100	90	0	0
ESM-MSA-MCMC	116	109	66	.61

Supplementary Data 1. Primer sequences used in this study. This file lists the primers used in cloning and NGS library prep. All other primers for subpool amplification are listed with specific genes and archived with Zenodo (https://zenodo.org/records/17637645).

REFERENCES

1. M. Blum, A. Andreeva, L. C. Florentino, S. R. Chuguransky, T. Grego, E. Hobbs, B. L. Pinto, A. Orr, T. Paysan-Lafosse, I. Ponamareva, G. A. Salazar, N. Bordin, P. Bork, A. Bridge, L. Colwell, J. Gough, D. H. Haft, I. Letunic, F. Llinares-López, A. Marchler-Bauer, L. Meng-Papaxanthos, H. Mi, D. A. Natale, C. A. Orengo, A. P. Pandurangan, D. Piovesan, C. Rivoire, C. J. A. Sigrist, N. Thanki, F. Thibaud-Nissen, P. D. Thomas, S. C. E. Tosatto, C. H. Wu, A. Bateman, InterPro: The protein sequence classification resource in 2025. *Nucleic Acids Res.* **53**, D444–D456 (2025).

2. M. Steinegger, J. Söding, Clustering huge protein sequence sets in linear time. *Nat. Commun.* **9**, 2542 (2018).

3. N. Prywes, N. R. Phillips, L. M. Oltrogge, S. Lindner, L. J. Taylor-Kearney, Y.-C. C. Tsai, B. de Pins, A. E. Cowan, H. A. Chang, R. Z. Wang, L. N. Hall, D. Bellieny-Rabelo, H. M. Nisonoff, R. F. Weissman, A. I. Flamholz, D. Ding, A. Y. Bhatt, O. Mueller-Cajar, P. M. Shih, R. Milo, D. F. Savage, A map of the RuBisCO biochemical landscape. *Nature* **638**, 823–828 (2025).

4. L. Gonzalez Somermeyer, A. Fleiss, A. S. Mishin, N. G. Bozhanova, A. A. Igolkina, J. Meiler, M.-E. Alaball Pujol, E. V. Putintseva, K. S. Sarkisyan, F. A. Kondrashov, Heterogeneity of the GFP fitness landscape and data-driven protein design. *eLife* **11**, e75842 (2022).

5. K. S. Sarkisyan, D. A. Bolotin, M. V. Meer, D. R. Usmanova, A. S. Mishin, G. V. Sharonov, D. N. Ivankov, N. G. Bozhanova, M. S. Baranov, O. Soylemez, N. S. Bogatyreva, P. K. Vlasov, E. S. Egorov, M. D. Logacheva, A. S. Kondrashov, D. M. Chudakov, E. V. Putintseva, I. Z. Mamedov, D. S. Tawfik, K. A. Lukyanov, F. A. Kondrashov, Local fitness landscape of the green fluorescent protein. *Nature* **533**, 397–401 (2016).

6. K. Tsuboyama, J. Dauparas, J. Chen, E. Laine, Y. Mohseni Behbahani, J. J. Weinstein, N. M. Mangan, S. Ovchinnikov, G. J. Rocklin, Mega-scale experimental analysis of protein folding stability in biology and design. *Nature* **620**, 434–444 (2023).

7. W. P. Russ, M. Figliuzzi, C. Stocker, P. Barrat-Charlaix, M. Socolich, P. Kast, D. Hilvert, R. Monasson, S. Cocco, M. Weigt, R. Ranganathan, An evolution-based model for designing chorismate mutase enzymes. *Science* **369**, 440–445 (2020).

8. D. Repecka, V. Jauniskis, L. Karpus, E. Rembeza, I. Rokaitis, J. Zrimec, S. Poviloniene, A. Laurynenas, S. Viknander, W. Abuajwa, O. Savolainen, R. Meskys, M. K. M. Engqvist, A. Zelezniak, Expanding functional protein sequence spaces using generative adversarial networks. *Nat. Mach. Intell.* **3**, 324–333 (2021).

9. T. Hayes, R. Rao, H. Akin, N. J. Sofroniew, D. Oktay, Z. Lin, R. Verkuil, V. Q. Tran, J. Deaton, M. Wiggert, R. Badkundri, I. Shafkat, J. Gong, A. Derry, R. S. Molina, N. Thomas, Y. A. Khan, C. Mishra, C. Kim, L. J. Bartie, M. Nemeth, P. D. Hsu, T. Sercu, S. Candido, A. Rives, Simulating 500 million years of evolution with a language model. *Science* **387**, 850–858 (2025).

10. R. A. Hughes, A. D. Ellington, Synthetic DNA synthesis and assembly: Putting the synthetic in synthetic biology. *Cold Spring Harb. Perspect. Biol.* **9**, a023812 (2017).

11. A. Y. Borovkov, A. V. Loskutov, M. D. Robida, K. M. Day, J. A. Cano, T. Le Olson, H. Patel, K. Brown, P. D. Hunter, K. F. Sykes, High-quality gene assembly directly from unpurified mixtures of microarray-synthesized oligonucleotides. *Nucleic Acids Res.* **38**, e180 (2010).

12. N. C. Tang, A. Chilkoti, Combinatorial codon scrambling enables scalable gene synthesis and amplification of repetitive proteins. *Nat. Mater.* **15**, 419–424 (2016).

13. N. Eroshenko, S. Kosuri, A. H. Marblestone, N. Conway, G. M. Church, Gene assembly from chip-synthesized oligonucleotides. *Curr. Protoc. Chem. Biol.* **2012**, ch110190 (2012).

14. S. Kosuri, N. Eroshenko, E. M. LeProust, M. Super, J. Way, J. B. Li, G. M. Church, Scalable gene synthesis by selective amplification of DNA pools from high-fidelity microchips. *Nat. Biotechnol.* **28**, 1295–1299 (2010).

15. J. C. Klein, M. J. Lajoie, J. J. Schwartz, E.-M. Strauch, J. Nelson, D. Baker, J. Shendure, Multiplex pairwise assembly of array-derived DNA oligonucleotides. *Nucleic Acids Res.* **44**, e43 (2016).

16. C. Plesa, A. M. Sidore, N. B. Lubock, D. Zhang, S. Kosuri, Multiplexed gene synthesis in emulsions for exploring protein functional landscapes. *Science* **359**, 343–347 (2018).

17. C. Engler, R. Kandzia, S. Marillonnet, A one pot, one step, precision cloning method with high throughput capability. *PLOS ONE* **3**, e3647 (2008).

18. J. M. Pryor, V. Potapov, K. Bilotti, N. Pokhrel, G. J. S. Lohman, Rapid 40 kb genome construction from 52 parts through data-optimized assembly design. *ACS Synth. Biol.* **11**, 2036–2042 (2022).

19. J. M. Pryor, V. Potapov, R. B. Kucera, K. Bilotti, E. J. Cantor, G. J. S. Lohman, Enabling one-pot Golden Gate assemblies of unprecedented complexity using data-optimized assembly design. *PLOS ONE* **15**, e0238592 (2020).

20. V. Potapov, J. L. Ong, R. B. Kucera, B. W. Langhorst, K. Bilotti, J. M. Pryor, E. J. Cantor, B. Canton, T. F. Knight, T. C. Evans Jr, G. J. S. Lohman, Comprehensive profiling of four base overhang ligation fidelity by T4 DNA ligase and application to DNA assembly. *ACS Synth. Biol.* **7**, 2665–2674 (2018).

21. D. Öling, O. Lan-Chow-Wing, A. Martella, S. Gilberto, J. Chi, E. Cooper, T. Edström, B. Peng, D. Sumner, F. Karlsson, P. Volkov, C. I. Webster, R. Roth, FRAGLER: A fragment recycler application enabling rapid and scalable modular DNA assembly. *ACS Synth. Biol.* **11**, 2229–2237 (2022).

22. S. K. Subramanian, W. P. Russ, R. Ranganathan, A set of experimentally validated, mutually orthogonal primers for combinatorially specifying genetic components. *Synth Biol* **3**, ysx008 (2018).

23. S. Lund, V. Potapov, S. R. Johnson, J. Buss, N. A. Tanner, Highly parallelized construction of DNA from low-cost oligonucleotide mixtures using data-optimized assembly design and Golden Gate. *ACS Synth. Biol.* **13**, 745–751 (2024).

24. E. A. Rodriguez, R. E. Campbell, J. Y. Lin, M. Z. Lin, A. Miyawaki, A. E. Palmer, X. Shu, J. Zhang, R. Y. Tsien, The growing and glowing toolbox of fluorescent and photoactive proteins. *Trends Biochem. Sci.* **42**, 111–129 (2017).

25. T. J. Lambert, FPbase: A community-editable fluorescent protein database. *Nat. Methods* **16**, 277–278 (2019).

26. The UniProt Consortium, UniProt: The Universal Protein Knowledgebase in 2025. *Nucleic Acids Res.* **53**, D609–D617 (2025).

27. K. K. Yang, N. Fusi, A. X. Lu, Convolutions are competitive with transformers for protein sequence pretraining. *Cell Syst.* **15**, 286–294.e2 (2024).

28. K. K. Yang, N. Zanichelli, H. Yeh, Masked inverse folding with sequence transfer for protein representation learning. *Protein Eng. Des. Sel.* **36**, gzad015 (2023).

29. R. Rao, J. Liu, R. Verkuil, J. Meier, J. F. Canny, P. Abbeel, T. Sercu, A. Rives, MSA transformer. bioRxiv 2021.02.12.430858 (2021); https://www.biorxiv.org/content/10.1101/2021.02.12.430858v1.

30. N. B. Lubock, D. Zhang, A. M. Sidore, G. M. Church, S. Kosuri, A systematic comparison of error correction enzymes by next-generation sequencing. *Nucleic Acids Res.* **45**, 9206–9217 (2017).

31. W. Fan, Y. Zhou, S. Wang, Y. Yan, H. Liu, Q. Zhao, L. Song, Q. Li, Computational Protein Science in the Era of Large Language Models (LLMs). arXiv:2501.10282 (2025); https://doi.org/10.48550/arXiv.2501.10282.

32. Z. Wu, K. E. Johnston, F. H. Arnold, K. K. Yang, Protein sequence design with deep generative models. *Curr. Opin. Chem. Biol.* **65**, 18–27 (2021).

33. D. H. Bryant, A. Bashir, S. Sinai, N. K. Jain, P. J. Ogden, P. F. Riley, G. M. Church, L. J. Colwell, E. D. Kelsic, Deep diversification of an AAV capsid protein by machine learning. *Nat. Biotechnol.* **39**, 691–696 (2021).

34. C. R. Freschlin, S. A. Fahlberg, P. Heinzelman, P. A. Romero, Neural network extrapolation to distant regions of the protein fitness landscape. *Nat. Commun.* **15**, 6405 (2024).

35. A. M. Sidore, C. Plesa, J. A. Samson, N. B. Lubock, S. Kosuri, DropSynth 2.0: High-fidelity multiplexed gene synthesis in emulsions. *Nucleic Acids Res.* **48**, e95 (2020).

36. H. Choi, Y. Choi, J. Choi, A. C. Lee, H. Yeom, J. Hyun, T. Ryu, S. Kwon, Purification of multiplex oligonucleotide libraries by synthesis and selection. *Nat. Biotechnol.* **40**, 47–53 (2022).

37. G. Boël, R. Letso, H. Neely, W. N. Price, K.-H. Wong, M. Su, J. D. Luff, M. Valecha, J. K. Everett, T. B. Acton, R. Xiao, G. T. Montelione, D. P. Aalberts, J. F. Hunt, Codon influence on protein expression in *E. coli* correlates with mRNA levels. *Nature* **529**, 358–363 (2016).

38. Q. Xu, M. R. Schlabach, G. J. Hannon, S. J. Elledge, Design of 240,000 orthogonal 25mer DNA barcode probes. *Proc. Natl. Acad. Sci.* **106**, 2289–2294 (2009).

39. S. R. Johnson, X. Fu, S. Viknander, C. Goldin, S. Monaco, A. Zelezniak, K. K. Yang, Computational scoring and experimental evaluation of enzymes generated by neural networks. *Nat. Biotechnol.* **43**, 396–405 (2025).

40. S. R. Johnson, S. Monaco, K. Massie, Z. Syed, Generating novel protein sequences using Gibbs sampling of masked language models. bioRxiv 2021.01.26.428322 (2021); https://www.biorxiv.org/content/10.1101/2021.01.26.428322v1.

41. K. S. Sarkisyan, O. A. Zlobovskaya, D. A. Gorbachev, N. G. Bozhanova, G. V. Sharonov, D. B. Staroverov, E. S. Egorov, A. V. Ryabova, K. M. Solntsev, A. S. Mishin, K. A. Lukyanov, KillerOrange, a genetically encoded photosensitizer activated by blue and green light. *PLOS ONE* **10**, e0145287 (2015).

42. R. C. Edgar, UPARSE: Highly accurate OTU sequences from microbial amplicon reads. *Nat. Methods* **10**, 996–998 (2013).

43. B. Townshend, A. B. Kennedy, J. S. Xiang, C. D. Smolke, High-throughput cellular RNA device engineering. *Nat. Methods* **12**, 989–994 (2015).

44. F. Pedregosa, G. Varoquaux, A. Gramfort, V. Michel, B. Thirion, O. Grisel, M. Blondel, P. Prettenhofer, R. Weiss, V. Dubourg, J. Vanderplas, A. Passos, D. Cournapeau, M. Brucher, M. Perrot, E. Duchesnay. Scikit-learn: Machine learning in Python. *J. Mach. Learn. Res.* **12**, 2825–2830 (2011).
